# Loss of Lepr in Fibroblasts Drives Tumor Development by Promoting Macrophage Infiltration

**DOI:** 10.1002/advs.78562

**Published:** 2026-10-31

**Authors:** Manyu Song, Wuqi Yang, Bangran Qi, Liyuan Hou, Xu Yang, Jiuzhi Xu, Shengyuan Tian, Shuiling Jin, Lu Yu, Zhengquan Yu, Min Deng, Jiaqiang Huang, Suoqin Jin, Cong Lv

**Affiliations:** ^1^ Key Laboratory of Precision Nutrition and Food Quality Department of Nutrition and Health China Agricultural University Beijing China; ^2^ Beijing Advanced Innovation Center for Food Nutrition and Human Health Department of Nutrition and Health China Agricultural University Beijing China; ^3^ State Key Laboratory of Animal Biotech Breeding College of Biological Sciences China Agricultural University Beijing China; ^4^ The First Affiliated Hospital of Zhengzhou University Zhengzhou Henan China; ^5^ Tianjian Laboratory of Advanced Biomedical Sciences Academy of Medical Sciences Zhengzhou University Zhengzhou Henan China; ^6^ School of Mathematics and Statistics Wuhan University Wuhan China

**Keywords:** colorectal cancer, Lepr^neg^Mmp^high^ fibroblasts, leptin receptor, macrophage infiltration, TNFα and TGFβ signaling pathways

## Abstract

Cancer‐associated fibroblasts (CAFs) are critical regulators of colorectal cancer (CRC) progression, yet the mechanisms governing their functional plasticity and state transitions remain incompletely understood. Here, we uncover a tumor‐driven fibroblast state transition in CRC characterized by loss of leptin receptor (Lepr) expression and acquisition of pro‐inflammatory features. We show that cooperative TNFα and TGFβ signaling drives this reprogramming, accompanied by repression of the Tcf21‐Lepr regulatory axis. Fibroblasts undergoing this transition acquire the capacity to promote macrophage recruitment and accelerate tumor development. Loss of Lepr or Tcf21 in fibroblasts upregulates Csf3, which contributes to the enhanced macrophage‐recruiting capacity of the reprogrammed fibroblasts. Consistently, blockade of TNFα and TGFβ signaling disrupts fibroblast reprogramming and attenuates macrophage infiltration. Together, our findings uncover how inflammatory signaling reshapes stromal cell states to promote macrophage accumulation, ultimately fostering a pro‐tumorigenic niche that accelerates CRC progression.

## Graphical abstract

TNFα and TGFβ signaling reprograms Lepr^+^ fibroblasts toward a Lepr^neg^Mmp^high^ inflammatory CAF (iCAF) state, with downregulation of the Tcf21‐Lepr axis contributing to this transition. Lepr loss induces Csf3, which contributes to the enhanced macrophage‐recruiting capacity of these iCAFs, thereby promoting tumor development. Thus, inflammatory signaling reshapes fibroblast states to establish a pro‐tumorigenic microenvironment in colorectal cancer.

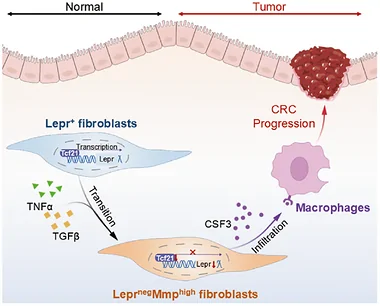

## 1 Introduction

Colorectal cancer (CRC) remains one of the leading causes of cancer‐related morbidity and mortality worldwide. Beyond malignant epithelial cells, tumor development is critically shaped by the tumor microenvironment (TME), a complex ecosystem composed of stromal, immune, and vascular components that collectively regulate tumor growth, immune evasion, and therapeutic response [1]. Among these, cancer‐associated fibroblasts (CAFs) represent a dominant and highly plastic stromal population that profoundly influences CRC biology [2]. CAFs support tumor development by secreting growth factors, remodeling the extracellular matrix, and shaping tissue architecture, while also exerting potent immunomodulatory effects through regulation of immune cell recruitment and activation [3, 4]. Despite their central role, the mechanisms governing CAF functional diversification in CRC remain incompletely understood.

Accumulating evidence indicates that CAFs are not uniform or static entities but exhibit considerable functional plasticity within the TME [5]. Single‐cell transcriptomic studies have revealed that fibroblasts adopt multiple transcriptional and functional states‐rather than fixed lineages‐in response to dynamic crosstalk with tumor‐ and immune‐derived cues [6]. These signals drive CAF differentiation into specialized subsets like the pro‐fibrotic myofibroblastic (myCAF) and pro‐inflammatory (iCAF) states, enabling CAFs to adaptively support tumor development through diverse mechanisms [7, 8]. However, the molecular mechanisms governing transitions between these distinct fibroblast states, as well as the intrinsic regulatory circuits and extrinsic signaling combinations that reinforce or disrupt these phenotypic states, remain incompletely understood. A critical gap persists in our knowledge of whether such signals act by suppressing homeostatic fibroblast programs that normally constrain inflammatory activation, and how intrinsic determinants dictate whether a fibroblast acquires pro‐tumorigenic functions or retains homeostatic features. Unraveling these questions is essential for deciphering stromal contributions to CRC biology and identifying novel targets for stromal reprogramming therapies.

Leptin receptor (Lepr) is expressed by stromal cells in multiple tissues, where Lepr^+^ fibroblasts are broadly distributed across organs including bone marrow, blood vessel, sympathetic nerves, and lungs, serving as key stromal cells for tissue homeostasis [9, 10, 11, 12]. They play pivotal roles in physiological processes such as hematopoietic support, vascular maintenance, and inflammatory regulation. Recent studies have extended the function of Lepr^+^ cells to the intestine. Our previous study demonstrated that intestinal Lepr^+^ fibroblasts contribute to epithelial homeostasis under physiological conditions and promote epithelial repair following injury. Furthermore, Lepr‐mediated leptin signaling in fibroblasts sustains intestinal stem and progenitor cell populations through a stromal IGF1‐epithelial IGF1R axis [13]. These findings suggest that Lepr‐expressing fibroblasts function as key stromal mediators linking systemic metabolic signals to local intestinal stem cell activity and tissue maintenance. It is possible that Lepr signaling also influences the functional states of fibroblasts under homeostatic conditions. Given the diverse roles of Lepr^+^ fibroblasts in maintaining intestinal homeostasis and coordinating stromal‐epithelial interactions, whether these cells contribute to CRC progression remains unclear. Moreover, it is unknown whether Lepr regulates fibroblast functional states within the TME.

In this study, we identify a tumor‐driven transition in which Lepr‐lineage fibroblasts acquire a pro‐inflammatory, matrix‐remodeling phenotype accompanied by reduced Lepr expression. Cooperative TNFα and TGFβ signaling promotes this fibroblast reprogramming through repression of the Tcf21‐Lepr regulatory axis, accompanied by acquisition of an enhanced macrophage‐recruiting program to which increased Csf3 production contributes, thereby fostering a tumor‐promoting microenvironment and promoting colorectal tumor development. Together, these findings reveal how inflammatory cues disrupt the fibroblast identity to establish a macrophage‐recruiting stromal niche that supports CRC progression.

## 2 Results

### 2.1 Lepr‐Lineage‐Derived Lepr^neg^Mmp^high^ Cells Are Enriched in Colon Tumors

To understand the role of Lepr‐lineage fibroblasts in CRC progression, we first established an AOM/DSS‐induced colon tumor mouse model in 8‐week‐old *Lepr‐Cre;mTmG* mice, allowing the labeling Lepr‐lineage cells by GFP (Figure S1A,B). Notably, the abundance of GFP^+^ cells significantly increased during the progression of CRC, which accounted for approximately 40% of Col1a1^+^ stromal cells in colorectal tumors (Figure 1A; Figure S1C). To dissect the cellular features of Lepr‐lineage cells, GFP^+^ cells from normal colon and colorectal tumor tissues in *Lepr‐Cre;mTmG* mice were isolated, and subjected to droplet‐based 3ʹ‐scRNA‐seq (Figure 1B; Figure S1D). The preprocessed scRNA‐seq data of normal colon and colorectal tumor tissues was computationally integrated and displayed high GFP transcript (Figure S1E,F). These cells were primarily clustered into fibroblasts (*Col1a2^high^* and *Rgs5^low^*), endothelial cells (*Vwf^high^*), lymphatic endothelial cells (*Lye1^high^*), smooth muscle (*Myl9^high^*), and pericytes (*Rgs5^high^*) (Figure S1G,H). To understand the contribution of *Lepr* ^+^ fibroblast lineages in CRC progression, we further performed subclustering analysis of these fibroblasts, leading to the identification of four distinct subtypes based on altered gene expressions (Figure 1C; Figure S1I). Strikingly, Fb1 and Fb2 clusters were primarily present in both normal tissues while Fb3 and Fb4 clusters were specifically presented in colorectal tumors (Figure 1C). We found that both Fb3 and Fb4 subsets shared elevated expression of factors such as *Cxcl14*, *Hp*, *Lcn2*, and *Clu*, which are broadly implicated in inflammatory responses. Notably, Fb4 exhibited a distinct transcriptional profile characterized by the upregulation of inflammation‐associated genes, including *Mmp3*, *Mmp13*, *Cxcl5*, *Il11*, *Il1rl1*, and *Inhba* (Figure 1D,E). These genes appear to function as iCAFs within the TME, closely associated with immune cell recruitment, inflammatory signaling, and tumor development. Moreover, *Lepr* expression was significantly lower in the Fb4, but not the Fb3 subset (Figure 1E). Consistently, RNAscope analysis showed that the proportion of GFP^+^ fibroblasts with detectable *Lepr* mRNA was reduced in colorectal tumors compared with normal colon (Figure S1J). These findings support a progressive loss of Lepr expression within the Lepr‐lineage fibroblast compartment during tumor‐associated fibroblast reprogramming. Thus, we refer to the Fb4 cluster as Lepr^neg^Mmp^high^ cells based on the expression of marker genes. To further characterize the fibroblast subsets in colon tumor, we performed gene set scoring analysis based on their gene expression levels. Notably, Lepr^neg^Mmp^high^ subset exhibited the highest activity in pathways related to wound healing [14], glycolysis [15], degradation of ECM [16], and inflammatory response [17], all of which have been well established to play critical roles in tumor development (Figure 1F). Although Fb3 cells are also enriched within tumor tissues, their pro‐tumorigenic pathway activities are lower than those of Lepr^neg^Mmp^high^ cells (Figure 1F). Together, these findings suggest that the Lepr^neg^Mmp^high^ subset represents a functionally specialized CAF population with strong pro‐tumor potential, whereas Fb3 cells may serve as a transitional state bridging homeostatic Lepr‐lineage fibroblasts in normal colon and tumor‐promoting Lepr^neg^Mmp^high^ fibroblasts in colon tumor. Immunofluorescence staining for Mmp3, Il1rl1 and GFP confirmed that Lepr^neg^Mmp^high^ cells exclusively present in colorectal tumor tissue, but not in normal colon (Figure 1G).

**FIGURE 1:**
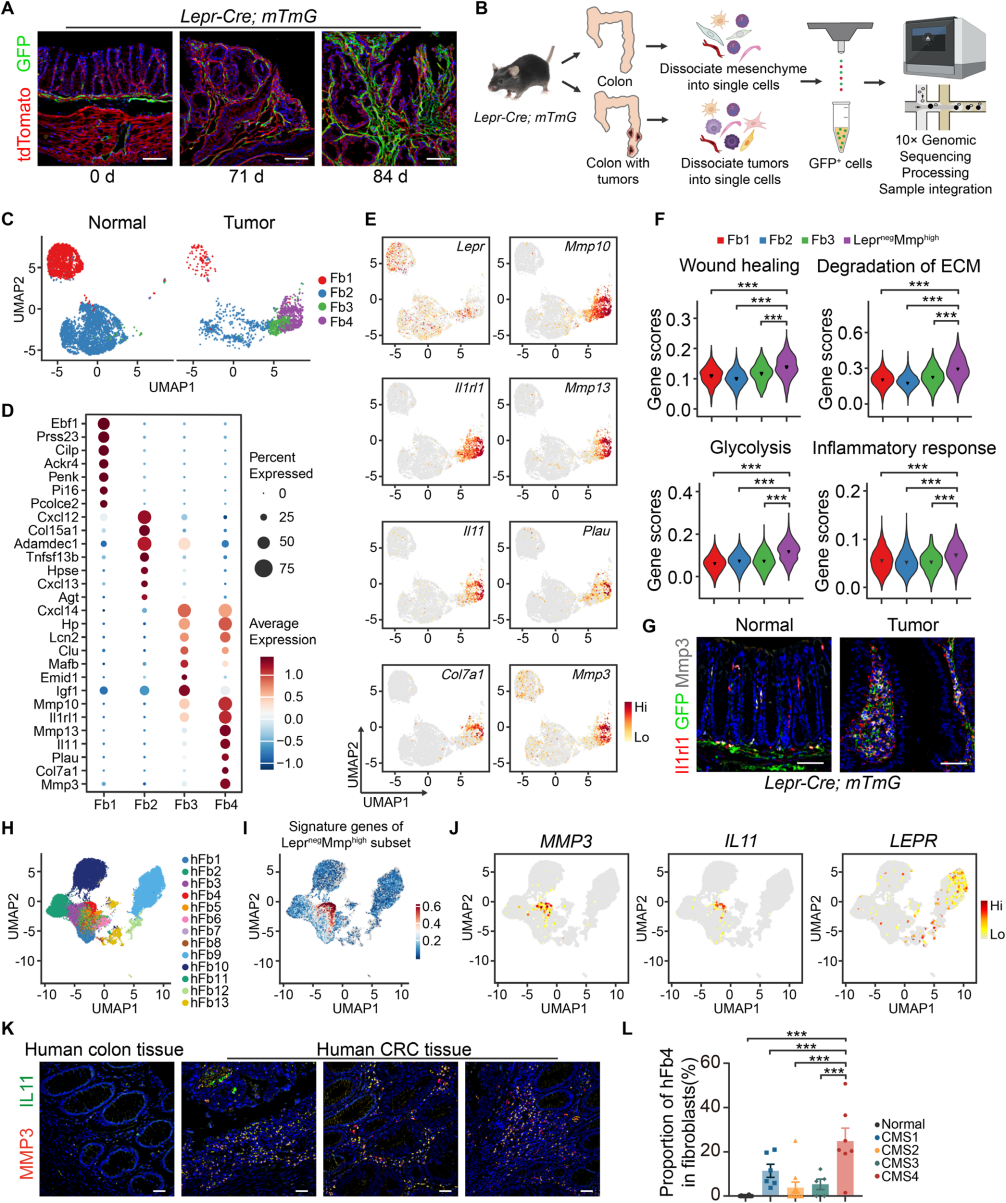
Lepr‐lineage fibroblast‐derived Lepr^neg^Mmp^high^ cells are enriched in colon tumors. (A) Representative fluorescence images for GFP (refers to Lepr reporter cells) and td‐Tomato in sections of colon and tumor from *Lepr‐Cre;mTmG* mice. n = 5 mice for each group. Scale bar, 100 µm. (B) Schematic diagram of GFP^+^ cells isolation from colon or tumor tissues and 3ʹ‐scRNA‐seq of GFP^+^ cells using droplet‐based device 10× Genomics. (C) UMAP plot revealing cellular heterogeneity of GFP^+^ fibroblasts from normal colon and tumor tissues of *Lepr‐Cre;mTmG* mice. Four distinct clusters are identified and annotated. (D) Dot plots displaying the expression patterns of signature genes for each fibroblast subset in panel C. (E) Feature plots of the identified marker genes in Fb4 subset based on normalized counts. Expression levels for each cell are color‐coded. Gray represents low expression and red represents high expression. (F) Gene set scoring for tumor development–related signaling pathways in each fibroblast subset in panel C. (G) Representative immunofluorescence images of GFP, Il1rl1, and Mmp3 staining of colon and tumor tissues from *Lepr‐Cre;mTmG* mice. n = 5 mice for each group. Scale bar, 50 µm. (H) UMAP plot revealing cellular heterogeneity of fibroblasts from the single‐cell atlas of human colorectum under both physiological and pathological conditions. (I) Gene set scoring showing enrichment of signature genes of Lepr^neg^Mmp^high^ subset in the human CRC fibroblast scRNA‐seq dataset  in panel H. (J) Feature plots of expression distribution for *MMP3*, *IL11* and *LEPR* in the human CRC fibroblasts scRNA‐seq dataset. Expression levels are color‐coded. (K) Representative Immunofluorescence images of MMP3 and IL11 in human colorectal paracancerous (n = 3) and tumor tissues from CRC patients (n = 7). Scale bar, 50 µm. (L) The proportion of hFb4 fibroblasts from the human CRC fibroblasts scRNA‐seq dataset that were stratified by CMS classifications. Data are presented as mean ± SEM (L). Wilcoxon Rank‐Sum test (F) and one‐way ANOVA followed by Tukey's multiple comparisons test (L) were used. ^*^ *p* < 0.05, and ^***^ *p* < 0.001. See also Figures S1–S3.

To determine whether the Lepr^neg^Mmp^high^ subset could also be identified within the broader fibroblast landscape of colorectal tumors, we analyzed a publicly available scRNA‐seq dataset from AOM/DSS‐induced mouse colorectal tumors (Figure S2A) [18]. After reclustering the fibroblast compartment, five transcriptionally distinct fibroblast populations were identified (Figure S2B). One subset exhibited prominent expression of the Lepr^neg^Mmp^high^ marker genes defined in our Lepr‐lineage dataset and was therefore designated as an Fb4‐like population (Figure S2C). The Fb4‐like population accounted for 18% of the fibroblast compartment in this dataset (Figure S2D). Comparison with established CAF‐state markers showed that this Fb4‐like population preferentially expressed multiple iCAF‐associated genes (Figure S2E). Gene‐set scoring revealed the strongest enrichment of the inflammatory response program in the Fb4‐like population (Figure S2F). These findings identify an Fb4‐like fibroblast population with prominent inflammatory CAF‐associated features within the complete fibroblast landscape of mouse colorectal tumors.

To investigate whether analogous cell populations exist in human CRC, fibroblasts were extracted from multiple single‐cell transcriptomic datasets including normal colorectal tissue, ulcerative colitis, polyps, adjacent normal tissue, and colorectal carcinomas (Figure S3A–C) [19], and a total of thirteen fibroblast subpopulations were identified (Figure 1H). AUCell scoring analysis was used to map the differentially expressed gene (DEG) signatures of the four murine fibroblast subsets onto the human fibroblast atlas, revealing subset‐specific enrichment patterns across the human fibroblast landscape (Figure 1I; Figure S3D). Notably, the DEGs of Lepr^neg^Mmp^high^ subset were specifically enriched in the human hFb4 fibroblast cluster, which is characterized by high expression of *MMP3*, *CXCL8*, *MMP1*, *CXCL5*, *CXCL1*, *IL24*, *CXCL6*, *IL11*, and *IL6*, accompanied by remarkable low expression of *LEPR* (Figure 1J; Figure S3E). It is noteworthy that hFb4 subset was predominantly found in colorectal carcinoma tissues, exhibited low abundance in inflamed ulcerative colitis samples and was rarely detected in other pathological or normal tissues (Figure S3F). Immunofluorescence staining for MMP3 and IL11 further confirmed that hFb4 subset was specifically localized within human colorectal tumor tissues, consistent with the observations in murine model (Figure 1K). These findings demonstrated the existence of a distinct fibroblast subpopulations in human CRC is closely aligning with the murine Lepr^neg^Mmp^high^ cells. To further investigate the clinical significance of the hFb4 subset in CRC, we analyzed its distribution across the consensus molecular subtypes (CMS). The results showed that hFb4 was most prominently enriched in CMS4, which is highly dependent on tumor microenvironmental cues and characterized by pronounced stromal activation and immunosuppressive signaling (Figure 1L) [20, 21]. This suggests that the hFb4 fibroblast subpopulation may play a key role in modulating tumor‐stroma interactions in aggressive disease.

### 2.2 Combined TNFα and TGFβ Promotes Lepr‐Lineage Fibroblast Reprogramming toward the Lepr^neg^Mmp^high^ State

To explore the transcriptional relationship among Lepr‐lineage fibroblast states, we performed pseudotemporal trajectory analysis to order GFP^+^ fibroblasts along an inferred trajectory based on their transcriptional profiles. The Pi16^+^ fibroblast subpopulation has been described as a quiescent fibroblast population with the potential to give rise to more specialized fibroblast states [22]. Because Pi16 was highly enriched in the Fb1 subpopulation, Fb1 was selected as the root of the trajectory (Figure 2A,B). Pseudotime analysis ordered fibroblasts along a continuum from Fb1 through Fb2 and Fb3 toward the Lepr^neg^Mmp^high^ state (Figure 2C,D). Consistent with this transcriptional ordering, Lepr expression progressively decreased along pseudotime (Figure 2E). These findings are consistent with a progressive transcriptional shift toward the Lepr^neg^Mmp^high^ state.

**FIGURE 2:**
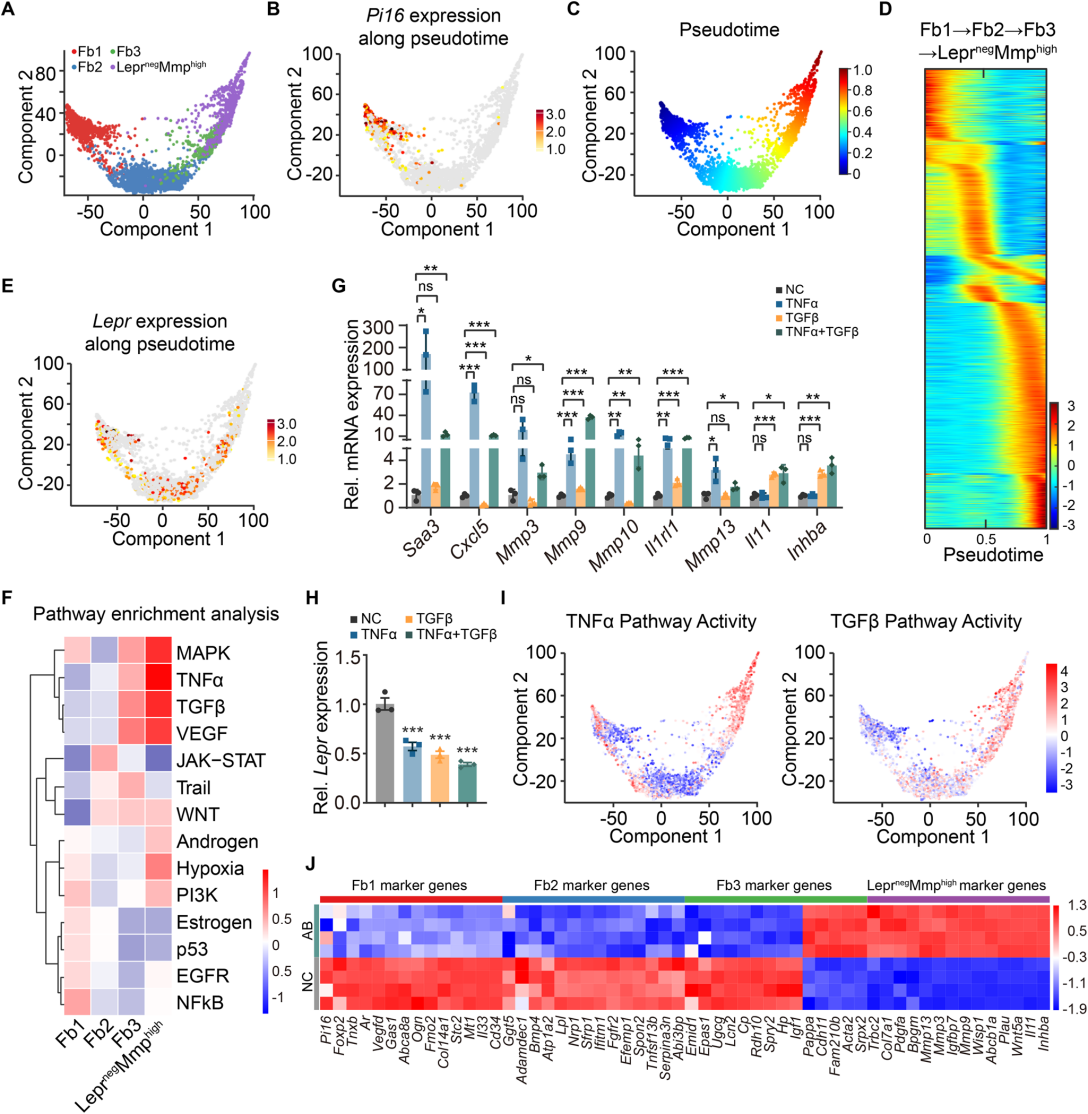
Combined TNFα and TGFβ promotes Lepr‐lineage fibroblast reprogramming toward the Lepr^neg^Mmp^high^ state. (A) PHATE visualization of GFP^+^ fibroblasts from colon and tumor of *Lepr‐Cre;mTmG* mice. (B) Feature plots of expression distribution for *Pi16*. Expression levels are color‐coded as in the PHATE space. (C) Pseudotemporal trajectory analysis with cells in panel A colored for their inferred pseudotime. (D) Changes in pseudotime‐dependent gene expression along the trajectory from Fb1 subset to Lepr^neg^Mmp^high^ subset. (E) Feature plots of expression distribution for *Lepr*. Expression levels are color‐coded as in the PHATE space. (F) The inferred pathway activity scores for each of the four fibroblast subsets. (G) GFP^+^ fibroblasts isolated from normal colon of *Lepr‐Cre;mTmG* mice were treated with conditioned medium (CM) supplemented with TNFα and/or TGFβ for 36 h. qRT‐PCR analysis of signature genes of Lepr^neg^Mmp^high^ fibroblast subset (n = 3). (H) qRT‐PCR analysis of *Lepr* in GFP^+^ fibroblasts (panel E) treated with TNFα and/or TGFβ for 36 h (n = 3). (I) The distributions of TNFα and TGFβ signaling activities along the fibroblast trajectory. (J) Heatmap depicting the altered marker genes of Fb1, Fb2, Fb3 and Lepr^neg^Mmp^high^ subsets in the transcriptome profile of GFP^+^ fibroblasts isolating from normal colon of *Lepr‐Cre;mTmG* mice treated with Vehicle (NC) or TNFα&TGFβ (AB) (n = 4). Data are presented as mean ± SEM (G,H). Unpaired t test (G,H) was used. ns, not significant; ^*^ *p* < 0.05, ^**^ *p* < 0.01, and ^***^ *p* < 0.001. See also Figure S4.

Next, we sought to address how Lepr‐lineage fibroblasts transition to Lepr^neg^Mmp^high^ state. PROGENy [23] was utilized to evaluate the signaling pathway activity from gene expression of different fibroblast subtypes. This approach led to the identification of top9 pathway, including MAPK, TNFα, TGFβ, VEGF, Androgen, Hypoxia, PI3K, EGFR, and NF‐kB, which were predicted to significantly upregulate the expression of signature genes in Lepr^neg^Mmp^high^ cells (Figure 2F). Since VEGF receptors and androgen receptor were not expressed in Fb3 and Lepr^neg^Mmp^high^ subpopulations (Figure S4A), NF‐κB, MAPK [24] and PI3K [25] pathways act downstream of TNFα signaling, we excluded these pathways from further analysis. Hypoxia is a common characteristic of TME that drives tumor development through multiple mechanisms, including the induction and activation of CAF [26]. Thus, GFP^+^ cells isolated from the normal colon of *Lepr‐Cre;mTmG* mice were exposed to hypoxic. However, the expression levels of Lepr^neg^Mmp^high^ cell markers were not upregulated significantly (Figure S4B), excluding hypoxia as a causative factor. We next investigated the role of EGFR signaling pathway in Lepr^neg^Mmp^high^ cell generation. Following isolation and culture, GFP^+^ cells were treated with the EGFR ligand Areg, TGFα or Hbegf. However, it failed to induce Lepr^neg^Mmp^high^ cells characteristics, suggesting that Lepr^neg^Mmp^high^ cell generation is not mediated by EGFR signaling pathway (Figure S4C). Then, we investigate the function of TNFα and TGFβ pathways in Lepr^neg^Mmp^high^ cell transition. Remarkably, the transition of Lepr‐lineage fibroblasts into Lepr^neg^Mmp^high^ state is only observed upon combined treatment with TNFα and TGFβ, which triggers elevated expression of *Mmps*, *Cxcl5*, *Saa3*, *Il1rl1*, *IL11* and *Inhba* (Figure 2G). Simultaneously, combined TNFα and TGFβ treatment leads to a significant downregulation of *Lepr* expression in Lepr^+^ fibroblasts (Figure 2H). Notably, the activities of TNFα and TGFβ pathways were significantly elevated along the cellular pseudotemporal trajectory (Figure 2I). To identify the cellular sources of TNFα and TGFβ and evaluate their potential signaling toward fibroblasts in colorectal tumors, we performed cell‐cell communication analyses using scRNA‐seq datasets from AOM/DSS‐induced mouse tumors and human CRC. In mouse tumors, *Tnf* expression was predominantly detected in myeloid populations, particularly macrophages and neutrophils, whereas *Tgfb1* was more broadly expressed across multiple cell types, including macrophages and T cells, while *Tgfb2* was predominantly expressed in fibroblast (Figure S4D). CellChat analysis further revealed significant Tnf‐Tnfrsf1a and Tgfb1 ‐(Acvr1+Tgfbr1) interactions involving fibroblasts (Figure S4E–H). A similar pattern was observed in human CRC, in which TNF was mainly expressed in macrophages, while TGFB1 was distributed across a broader range of cell populations. Consistently, TNF‐TNFRSF1A and TGFβ1‐(ACVR1+TGFBR1) interactions with fibroblasts were also significantly enriched in the human dataset (Figure S4I–M). Together, these cross‐species analyses suggest that myeloid‐derived TNFα and TGFβ produced by multiple stromal and immune populations may contribute to fibroblast reprogramming within the colorectal tumor microenvironment. To further validate the role of TNFα and TGFβ co‐stimulation in driving the formation of the Lepr^neg^Mmp^high^ fibroblast subset, we performed bulk RNA‐seq on GFP^+^ fibroblasts from untreated controls (NC group) and TNFα&TGFβ co‐treated cells (AB group). Transcriptomic analysis revealed that marker genes of the Fb1 and Fb2 subsets were markedly downregulated, Fb3‐associated markers showed partial upregulation, whereas signature genes of the Lepr^neg^Mmp^high^ subset were significantly upregulated (Figure 2J). Collectively, these data suggest that TNFα and TGFβ are critical regulators required for the transition from Lepr^+^ fibroblasts to Lepr^neg^Mmp^high^ cells.

### 2.3 Lepr^neg^Mmp^high^ Cells Promotes Tumor Development by Promoting Macrophage Infiltration

To investigate the role of Lepr^neg^Mmp^high^ cells during tumor development, GFP^+^ fibroblasts were isolated from *Lepr‐Cre;mTmG* mice, pretreated with or without TNFα and TGFβ, and co‐injected with MC38 colorectal cancer cells into the subserosal layer of the cecum in C57BL/6J mice (Figure 3A). Strikingly, combined TNFα and TGFβ treatment significantly increased tumor size (Figure 3B–C), indicating that Lepr^neg^Mmp^high^ cells promote tumor progression. To further assess whether Lepr^neg^Mmp^high^ cells influence tumor molecular subtypes, RNA‐seq was performed on orthotopic tumors co‐injected with MC38 cells and GFP^+^ fibroblasts isolated from *Lepr‐Cre;mTmG* mice. Transcriptomic analysis revealed that, compared with controls, tumors in the TNFα and TGFβ‐treated group displayed significantly higher module scores for CMS4‐associated gene signatures, whereas no enrichment was observed for CMS1‐, CMS2‐, or CMS3‐associated markers (Figure 3D; Figure S5A). These results suggest that the presence of Lepr^neg^Mmp^high^ fibroblasts may drive colorectal tumors toward a the CMS4‐like transcriptional program.

**FIGURE 3:**
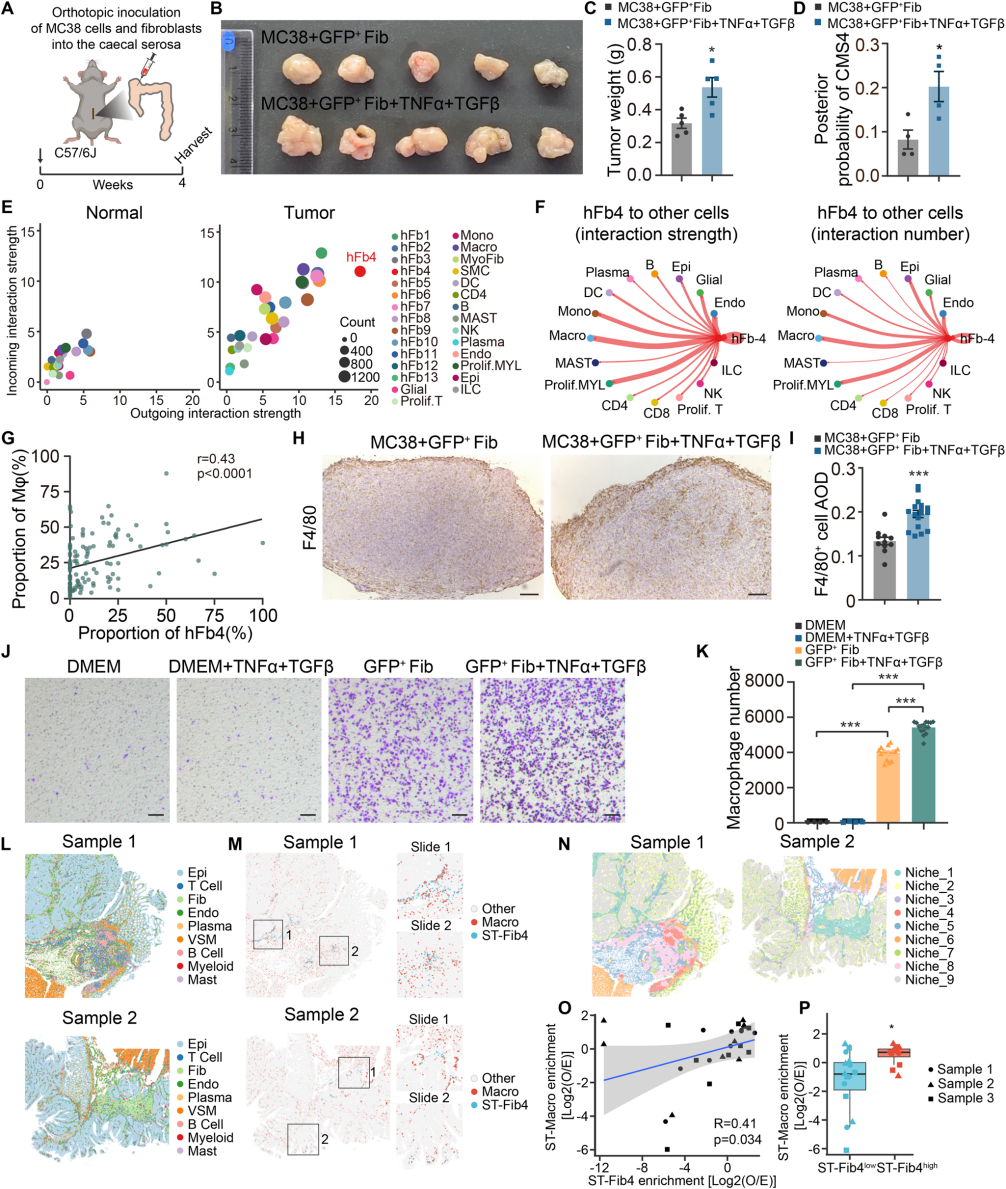
Lepr^neg^Mmp^high^ fibroblasts promotes tumor development by promoting macrophage infiltration. (A) Schematic diagram illustrating orthotopic co‐injection of GFP^+^ fibroblasts isolated from *Lepr‐Cre;mTmG* mice with MC38 cells into the cecal subserosa of 6‐week‐old C57BL/6J mice. (B) MC38 cells and GFP^+^ fibroblasts isolated from *Lepr‐Cre;mTmG* mice with or without TNFα/TGFβ treatment were injected into the cecum of 6‐week‐old C57BL/6J mice. Gross images of tumors collected 30 days after injection. (C) Quantification of tumor weight in panel B. (D) The module scores of CMS4 marker genes in different groups in panel B. n = 4 mice for each group. (E) Comparison of the outgoing (x‐axis) and incoming (y‐axis) interaction strength of each cell population between normal colon and tumor tissues, with circle size representing the number of significant interactions associated with different cell populations. (F) Circle plots showing the interaction strength and number of significant ligand‐receptor pairs between the hFb4 subset and other cell subpopulations, including epithelial cells, endothelial cells, immune cells, and glial cells. Edge width is proportional to the interaction number or strength, and the loops indicate autocrine signaling within the hFb4 subset. (G) Correlation between hFb4 abundance in fibroblasts and macrophage frequency within the immune cells in the single‐cell dataset shown in Figure S3B. (H) Immunohistochemical staining for F4/80 in tumors of mice in panel B. Scale bar, 100 µm. (I) Quantification of average optical density (AOD) of F4/80^+^ cells (Integrated option density/Tumor area). n = 5 mice for each group. (J) Transwell assay showing migration of macrophages induced by the supernatants from GFP^+^ fibroblasts with or without TNFα&TGFβ treatment. n = 4 independent experiments. Scale bar, 100 µm. (K) Quantification of cell migration in panel J. (L) Spatial distribution of major cell populations in two representative human CRC tumor sections. (M) Spatial distribution of ST‐Fib4 fibroblasts and ST‐Macro macrophages in two representative human CRC tumor sections. Boxed regions are shown at higher magnification below. (N) Spatial distribution of the identified cellular niches in two representative human CRC tumor sections. (O) Correlation analysis between the observed‐to‐expected (O/E) enrichment of ST‐Fib4 fibroblasts and ST‐Macro macrophages across spatial niches. Shapes indicate individual samples. (P) Comparison of ST‐Macro O/E enrichment between ST‐Fib4^low^ and ST‐Fib4^high^ spatial niches, stratified according to the median ST‐Fib4 O/E value. Data are presented as mean ± SEM (C, D, I and K). Unpaired t test (C, D, and I) and Welch ANOVA test (K) was used. ns, not significant, ^*^ *p* < 0.05, ^**^ *p* < 0.01, and ^***^ *p* < 0.001. See also Figures S5–S8.

Next, we sought to elucidate the mechanisms underlying their tumor‐promoting activity. CellChat was utilized to investigate potential cell‐cell communication between Fb4‐like fibroblast subpopulation and other cell types in AOM/DSS mouse CRC scRNA‐seq dataset. Fb4‐like fibroblasts exhibited high incoming and outgoing interaction strengths with macrophages, as well as epithelial cells and endothelial cells (Figure S5B,C). We also examined the corresponding hFb4 population in human CRC. Consistently, hFb4 fibroblasts exhibited strong outgoing signaling, with macrophages showing the most prominent predicted interactions in terms of both interaction number and strength (Figure 3E,F). Moreover, analysis of single‐cell datasets from human CRC revealed a significant positive correlation between hFb4 abundance and macrophage infiltration (Figure 3G). Orthotopic co‐injection assays demonstrated that TNFα and TGFβ induced GFP^+^ fibroblasts markedly increased F4/80^+^ macrophage infiltration within tumors (Figure 3H,I). To assess the direct effect of Lepr^neg^Mmp^high^ cells on macrophage, transwell migration assays were conducted using fibroblast‐derived conditioned media (CM). Notably, CM from GFP^+^ fibroblasts significantly promoted macrophage migration, and the effect further amplified by CM from TNFα and TGFβ co‐stimulated GFP^+^ fibroblasts (Figure 3J,K). Together, these findings indicate that TNFα&TGFβ‐induced fibroblast reprogramming enhances the macrophage‐recruiting capacity of GFP^+^ fibroblasts.

To further examine whether the association between Lepr^neg^Mmp^high^ fibroblasts and macrophages is spatially evident in human CRC, we analyzed an independent human CRC spatial transcriptomic dataset. After annotation of major cell populations (Figure 3L; Figure S5D), fibroblasts were reclustered into six subpopulations, among which ST‐Fib4 exhibited expression features resembling the Lepr^neg^Mmp^high^ /hFb4 fibroblast state (Figure S5E,F). Independent reclustering of the myeloid compartment identified macrophage, monocyte, and dendritic cell populations (Figure S5G,H). Spatial mapping revealed that frequent colocalization of ST‐Fib4 fibroblasts and macrophages within the same tumor regions (Figure 3M). Consistently, spatial niche analysis revealed a significant positive correlation between ST‐Fib4 and macrophage enrichment, with significantly greater macrophage enrichment in ST‐Fib4‐high niches than in ST‐Fib4‐low niches (Figure 3N–P).

Given the heterogeneous nature of tumor‐associated macrophages, the association between hFb4 abundance and specific macrophage states in human CRC was next investigated. Macrophages from the human CRC scRNA‐seq dataset were reclustered into eight transcriptionally distinct subpopulations (Figure S6A,B). Among these, the relative abundance of Macro_C3 showed a significant positive correlation with hFb4 abundance and was increased in hFb4high tumors (Figure S6C,D). Macro_C3 exhibited a prominent inflammatory/myeloid activation program, with enrichment of TNF, NF‐κB, IL‐17, Toll‐like receptor, and HIF‐1 signaling pathways (Figure S6E,F). These findings suggest that hFb4‐rich tumors are associated with selective enrichment of an inflammation‐associated TAM state rather than a uniform increase in all macrophage populations.

To further investigate the spatial relationship between hFb4 fibroblasts and the inflammation‐associated Macro_C3 subpopulation, we reclustered macrophages in the human CRC spatial transcriptomic dataset and identified an ST‐Macro_C3 subset exhibiting transcriptional features resembling those of Macro_C3 subset (Figure S7A,B). Spatial mapping revealed that ST‐Macro_C3 macrophages were frequently located near ST‐Fib4 fibroblasts (Figure S7C). Spatial proximity analysis further showed that the proportion of ST‐Macro_C3 macrophages located within 50 µm of ST‐Fib4 fibroblasts significantly higher than the expectation derived from permutation analysis in all three CRC samples (Figure S7D,E). Furthermore, spatial niche analysis revealed a positive correlation between ST‐Fib4 and ST‐Macro_C3 enrichment, with greater ST‐Macro_C3 enrichment in ST‐Fib4^high^ niches (Figure S7F–H). Together, these analyses provide spatial evidence supporting the association between hFb4 fibroblasts and Macro_C3‐associated macrophages in human CRC.

Considering that combined TNFα and TGFβ signaling drives the transition of Lepr‐lineage fibroblasts into Lepr^neg^Mmp^high^ state, we sought to ask whether blocking these pathways could disrupt fibroblast transition and attenuate CRC progression. To test this hypothesis, we employed the AOM/DSS‐induced colorectal tumors model in *Lepr‐Cre;mTmG* mice, animals were treated with TNFα and TGFβ inhibitors (AB‐Inh) every 3 days for a total of six doses during late‐stage tumorigenesis (Figure S8A). Compared to controls, administration with AB‐Inh markedly reduced tumor number (Figure S8B–D), accompanied by decreased the number of Lepr^neg^Mmp^high^ cells and diminished macrophage infiltration (Figure S8E,F). These findings indicate that systemic inhibition of TNFα and TGFβ signaling was associated with suppression of the Lepr^neg^Mmp^high^ fibroblast state and reduced macrophage accumulation in vivo.

### 2.4 Loss of *Lepr* Contributes to the Promoting Effects of Lepr^neg^Mmp^high^ Cell‐Induced Macrophage Infiltration via Upregulation of *Csf3*

scRNA‐seq analyses revealed a significant decrease in both *Lepr* ^+^ cell frequency and *Lepr* expression during tumor development, particularly within the Lepr^neg^Mmp^high^ cell subpopulation (Figure 4A). TCGA analysis confirmed conserved *Lepr* suppression in human colorectal tumor tissues (Figure 4B). This prompted us to test whether *Lepr* loss functionally contributes to the pro‐tumorigenic role of Lepr^neg^Mmp^high^ cells. Thus, we generated *Lepr* conditional knockout *Pdgfra^CreERT2^;Lepr^fl/fl^* mice (*Lepr^cko^*), in which *Lepr* is specifically deleted in fibroblasts upon Tamoxifen inducing (Figure S9A–C). Deletion of *Lepr* in fibroblasts promoted tumor development, as evidenced by increased tumor number, reflecting enhanced tumor initiation, and increased tumor size, reflecting enhanced tumor growth (Figure 4C–F). Considering that Lepr^neg^Mmp^high^ cells accelerate tumor development by enhancing macrophage infiltration, we asked whether *Lepr* deficiency in fibroblasts similarly orchestrates this pro‐tumorigenic mechanism. We observed that tumors from *Lepr* ^cko^ mice exhibited a significant increase in both the number and proportion of macrophages (Figure 4G–J). However, *Lepr* loss did not affect macrophage polarization, as tumors from both *Lepr^con^* and *Lepr* ^cko^ mice were predominantly infiltrated by M2 macrophages (Figure S9D). To determine whether *Lepr*‐deficient fibroblasts directly promote macrophage infiltration, we isolated GFP^+^ fibroblasts from *Lepr‐Cre;mTmG* mice and established primary cultures. CM from *Lepr*‐knockdown fibroblasts markedly enhanced macrophage infiltration compared to control medium (Figure 4K,L), indicating that *Lepr* loss in fibroblasts promotes macrophage recruitment via a paracrine mechanism. To evaluate whether the increased tumor burden observed in *Lepr* ^cko^ mice is macrophage‐dependent, we administered clodronate liposomes to deplete macrophages during AOM/DSS‐induced tumorigenesis (Figure 4M; Figure S9E). Notably, macrophage depletion reversed the increased tumor size in *Lepr* ^cko^ mice, consistent with reduced tumor growth, and showed a trend toward reducing tumor number (Figure 4N–P), suggesting that macrophages contribute to overall tumor development. These findings demonstrating that macrophages are critical mediators of the pro‐tumorigenic effects conferred by fibroblast *Lepr* loss.

**FIGURE 4:**
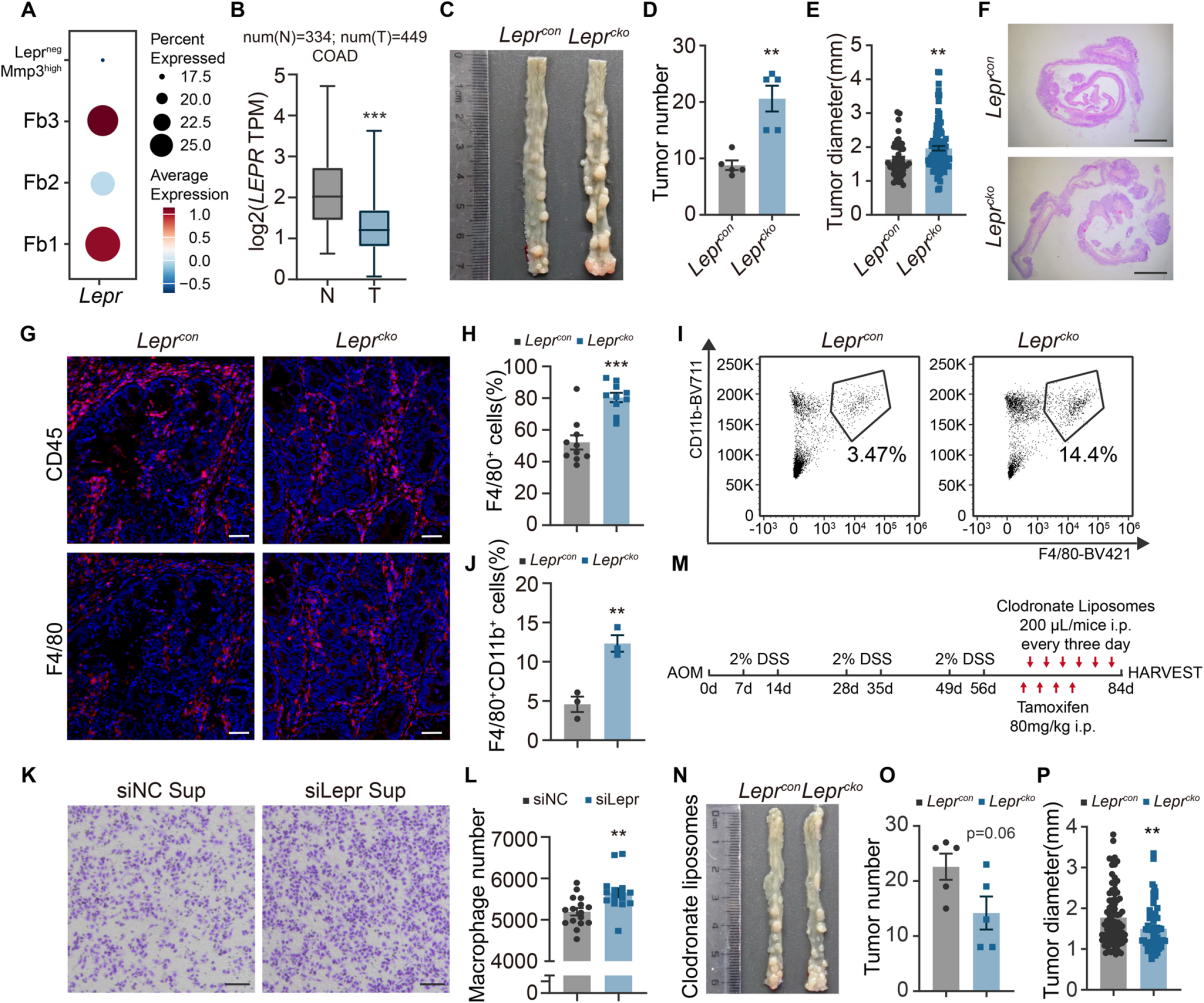
Loss of Lepr contributes to the promoting effects of Lepr^neg^Mmp^high^ fibroblast‐induced macrophage infiltration. (A) Percentage and average expression level of *Lepr* in Fb1, Fb2, Fb3 and *Lepr^neg^Mmp^high^* fibroblast subsets. (B) TCGA database analysis showed that *LEPR* expression was downregulated in COAD (Colon adenocarcinoma). Box plots represent the 25th and 75th percentiles, black line indicates median, and whiskers represent extremes. num: number of patient samples in normal colon (N) and tumor (T) tissues. (C) Representative gross images of AOM‐DSS mouse colon tumors from control (*Lepr^con^*) and *Pdgfrα* ^CreERT^;*Lepr* ^fl/fl^ (*Lepr^cko^*) mice. n = 5 mice for each group. (D) Quantification of the number of tumors from *Lepr^con^* and *Lepr^cko^* mice in panel C. (E) Quantification of the diameter of tumors from *Lepr^con^* and *Lepr^cko^* mice in panel C. *Lepr^con^*: n = 44 tumors from 5 mice; *Lepr^cko^*: n = 103 tumors from 5 mice. (F) Representative histological images of AOM‐DSS colon tumors from *Lepr^con^* and *Lepr^cko^* mice. n = 5 mice for each group. Scale bar, 2 mm. (G) Representative Immunofluorescence images of CD45 and F4/80 in colon tumors from *Lepr^con^* and *Lepr^cko^* mice. n = 5 mice for each group. Scale bar, 50 µm. (H) Quantification of the percentage of F4/80^+^ cells within the CD45^+^ cells in panel G. (I) Flow cytometry analysis of F4/80^+^CD11b^+^ cells in tumor tissues from *Lepr^con^* and *Lepr^cko^* mice. n = 3 mice for each group. (J) Quantification of the percentage of F4/80^+^&CD11b^+^ cells in panel I. (K) Transwell assay showing migration of macrophages induced by the supernatants from GFP^+^ fibroblasts with siNC or siLepr treatment. n = 4 independent experiments. Scale bar, 100 µm. (L) Quantification of cell migration in panel K. (M) Schematic diagram of AOM/DSS‐induced colon tumor mouse model with Tamoxifen and clodronate liposomes administration. (N) Representative gross images of AOM‐DSS colon tumors from *Lepr^con^* and *Lepr^cko^* mice treated with clodronate liposome. n = 5 mice for each group. (O) Quantification of the number of tumors from *Lepr^con^* and *Lepr^cko^* mice in panel N. (P) Quantification of the diameter of tumors from *Lepr^con^* and *Lepr^cko^* mice in panel N. *Lepr^con^*: n = 87 tumors from 5 mice; *Lepr^cko^*: n = 61 tumors from 5 mice. Data are presented as mean ± SEM (D,E,H,J,L,O,P). Unpaired t test (B,D,J,L,O) and Mann–Whitney test (E,H,P) were used. ^*^ *p* < 0.05, ^**^ *p* < 0.01, and ^***^ *p* < 0.001. See also Figure S9.

Given that Lepr^neg^Mmp^high^ fibroblasts robustly promote macrophage infiltration in tumors, and that fibroblast‐specific loss of *Lepr* similarly enhances macrophage accumulation, we next sought to delineate the downstream mediators underlying this macrophage‐recruiting activity. GFP^+^ fibroblasts isolated from *Lepr‐Cre;mTmG* mice were subjected to Lepr knockdown followed by RNA sequencing. Differential expression analysis revealed a distinct set of genes significantly upregulated upon *Lepr* silencing (Figure 5A). To prioritize candidate mediators potentially linked to the macrophage‐recruiting phenotype of Lepr^neg^Mmp^high^ fibroblasts, we intersected genes upregulated after *Lepr* knockdown with marker genes enriched in the Lepr^neg^Mmp^high^ fibroblast subset identified in identified by single‐cell analysis.

**FIGURE 5:**
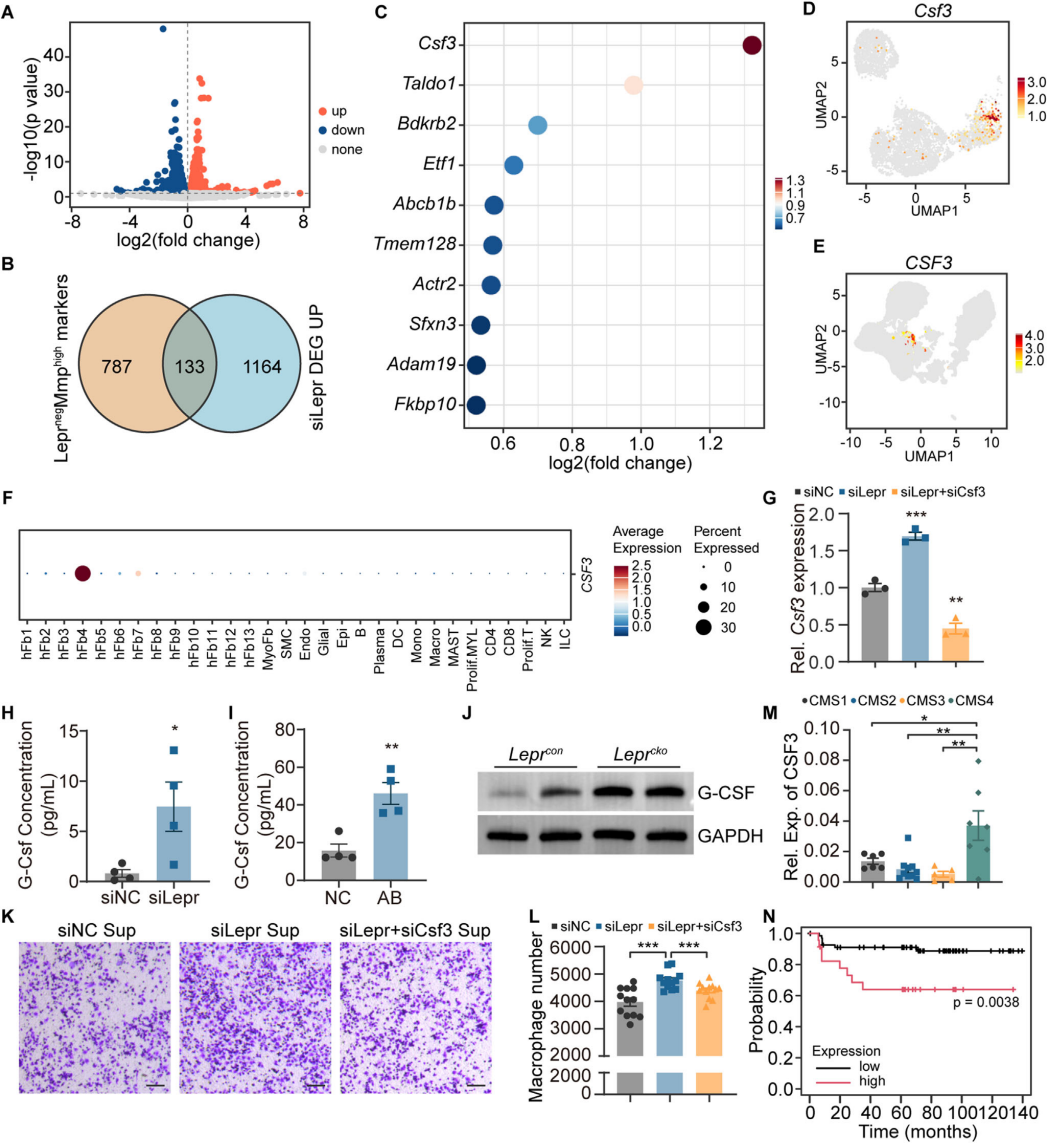
Csf3 links *Lepr* loss to macrophage recruitment in Lepr^neg^Mmp^high^ fibroblasts. (A) Volcano plot showing differentially expressed genes (DEGs) in GPF^+^ fibroblasts following Lepr knockdown (siLepr) compared with control (siNC). Red dots indicate significantly upregulated genes, blue dots indicate downregulated genes, and gray dots indicate non‐significant genes. (B) Venn diagram showing the overlap between upregulated DEGs upon Lepr knockdown and Lepr^neg^Mmp^high^ signature genes identified from the GFP^+^ fibroblast  scRNA‐seq analysis. (C) Dot plot of overlapping top 10 genes identified in panel B, ranked by fold change. (D) Feature plots of expression distribution for *Csf3* in the GFP^+^ fibroblasts in the murine fibroblast scRNA‐seq dataset (Figure S1I). (E) Feature plots of expression distribution for *CSF3* in the human CRC fibroblast scRNA‐seq dataset (Figure 1H). (F) Dot plot showing CSF3 expression across major cell populations in the human CRC scRNA‐seqN dataset. Dot size represents the percentage of cells expressing CSF3, and color indicates average expression level. (G) qRT‐PCR analysis for *Csf3* in GFP^+^ fibroblasts treated with siNC, siLepr and siLepr&siCsf3 for 48 h (n = 3). (H) ELISA quantification of G‐CSF in conditioned medium from GFP^+^ fibroblasts transfected with control siRNA (siNC) or Lepr siRNA (siLepr). n = 4 for each group. (I) ELISA quantification of G‐CSF in conditioned medium from GFP^+^ fibroblasts treated with vehicle (NC) or TNFα&TGFβ (AB). n = 4 for each group. (J) Western blotting for G‐CSF in AOM/DSS treated tumor from *Lepr^con^* and *Lepr^cko^* mice. GAPDH was used as a loading control. (K) Transwell assay showing migration of macrophages induced by the supernatants from GFP^+^ fibroblasts with siNC, siLepr or siLepr&siCsf3 treatment (n = 4). Scale bar, 100 µm. (L) Quantification of cell migration in panel K. (M) The relative expression of CSF3 in human normal colon and CRC tissues that were stratified by CMS classifications. (N) Kaplan–Meier survival curve of 91 CRC patients. p = 0.0038. Data are presented as mean ± SEM (G–I,L,M). Unpaired t test (G–I) was used. Brown‐Forsythe and Welch ANOVA test (L) was used. one‐way ANOVA followed by Tukey's multiple comparisons test (M) was used. ^*^ *p* < 0.05, ^**^ *p* < 0.01, and ^***^ *p* < 0.001. See also Figure S9.

This comparison yielded 113 shared genes (Figure 5B). Among these candidates, Csf3, which encodes granulocyte colony‐stimulating factor (G‐CSF), a cytokine with established roles in myeloid cell mobilization and functional modulation [27], showed the most pronounced upregulation following Lepr knockdown (Figure 5C), positioning it as a strong candidate downstream effector mediating fibroblast‐driven macrophage recruitment. Consistent with this notion, single‐cell transcriptomic analysis revealed that Csf3 expression was highly enriched in the Lepr^neg^Mmp^high^ subset in mice (Figure 5D) and predominantly localized to the analogous hFb4 fibroblast population in human CRC (Figure 5E,F).

Notably, Lepr knockdown markedly induced Csf3 expression in fibroblasts, whereas simultaneous Csf3 silencing effectively suppressed its expression (Figure 5G; Figure S9F). We next examined whether these transcriptional changes were accompanied by increased Csf3 protein (G‐CSF) production. ELISA analysis showed that *Lepr* knockdown significantly increased G‐CSF secretion from GFP^+^ fibroblasts isolated from normal colorectal tissue (Figure 5H). Consistent with the upstream fibroblast‐reprogramming model, combined TNFα and TGFβ treatment also markedly increased G‐CSF secretion from these fibroblasts (Figure 5I). Moreover, western blot analysis of AOM/DSS‐induced colorectal tumors revealed significantly higher G‐CSF protein levels in fibroblast‐specific Lepr^cko^ mice than in Lepr^con^ mice (Figure 5J), providing in vivo support for increased G‐CSF production associated with fibroblast Lepr loss. To test whether Csf3 functionally mediates the macrophage infiltration driven by the loss of Lepr expression, macrophages were treated with supernatant from the conditioned media of GFP^+^ fibroblasts isolated from *Lepr‐cre;mTmG* mice. Conditioned media from *Lepr* knockdown fibroblasts significantly enhanced macrophage migration, whereas dual inhibition of Lepr and Csf3 reversed the enhanced macrophage infiltration phenotype (Figure 5K,L), confirming that Lepr loss promotes Lepr^neg^Mmp^high^ cell‐mediated macrophage recruitment infiltration via upregulation of Csf3. Human CRC datasets analysis further revealed that CSF3 is highly expressed in CMS4 tumors (Figure 5M), in line with the enrichment of the hFb4 fibroblast in this tumor subtype. Moreover, CSF3 levels correlated with poor survival probability in CRC patients from GEO dataset (Figure 5N). Together, these findings indicate that Lepr loss increases Csf3 production in fibroblasts and that Csf3 contributes to the enhanced macrophage‐recruiting capacity associated with Lepr deficiency. These results support Csf3 as one functional component of the broader macrophage‐interacting program induced during fibroblast reprogramming.

### 2.5 The Loss of *Lepr* in Lepr^neg^Mmp^high^ Cells are Caused by TNFα and TGFβ‐Induced Tcf21 Downregulation

To identify the upstream regulators of Lepr, we examined transcription factor alterations in single‐cell data of GFP^+^ fibroblasts isolated from *Lepr‐cre;mTmG* mice. We systematically screened and ranked downregulated transcription factors in Lepr^neg^Mmp^high^ cells based on their gene expression levels (Figure 6A). Among these, we focused on Tcf21, a basic helix‐loop‐helix (bHLH) transcription factor known for its pivotal roles in mesoderm‐derived tissues, particularly in fibroblasts, smooth muscle cells and mesenchymal cells [28]. Tcf21 is recognized for maintaining fibroblast quiescence and functioning as a tumor suppressor [29, 30, 31]. Its expression was significantly depleted in both murine Lepr^neg^Mmp^high^ cells and human hFb4 cells (Figure 6B,C). Moreover, *Tcf21* expression positively correlated with *Lepr* levels in colon tissue, exhibiting a similar expression pattern to *Lepr* (Figure 6D). Knockdown of *Tcf21* in GFP^+^ fibroblasts isolated from *Lepr‐cre;mTmG* mice significantly downregulated *Lepr* expression, concomitant with decreased *Csf3* levels and increased macrophage infiltration (Figure 6E–G; Figure S9G). To further understand whether Tcf21 directly regulates *Lepr* expression, we analyzed the potential binding sites of transcription factors in the 2 kb region upstream of the transcription start site (TSS) of *Lepr* gene locus using the JASPAR database and identified two Tcf21‐binding sites (Figure 6H). Mutating two Tcf21‐binding sites markedly downregulated *Lepr* promoter luciferase activity (Figure 6I). We next performed CUT&RUN‐qPCR to assess Tcf21 occupancy at the *Lepr* promoter. Two predicted Tcf21‐binding regions within the Lepr promoter showed significant enrichment of Tcf21 compared with the IgG control (Figure 6J), providing direct evidence of Tcf21 binding to the *Lepr* promoter. Together, these results demonstrate that Tcf21 directly binds to and transcriptionally activates the *Lepr* promoter.

**FIGURE 6:**
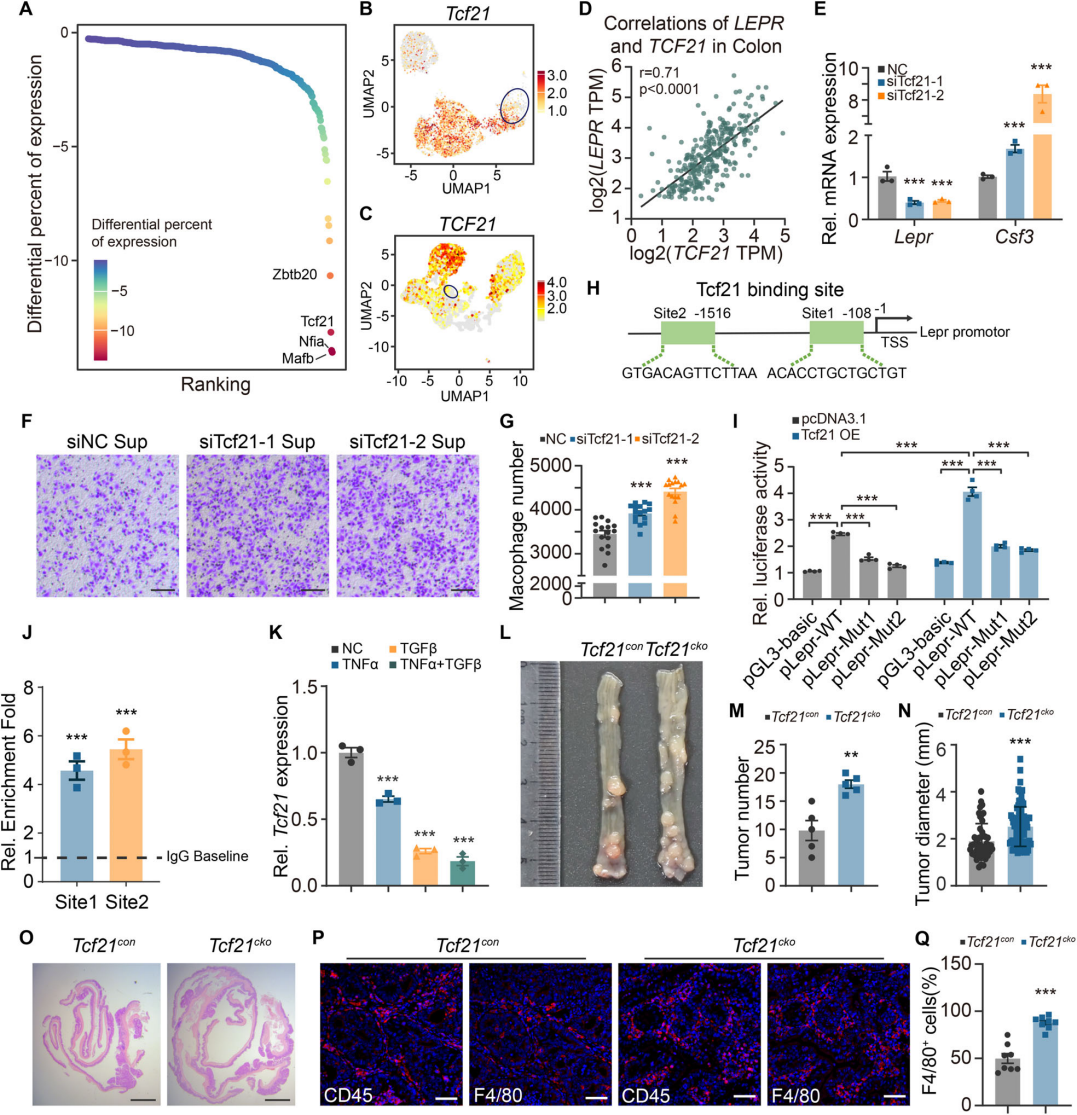
The loss of Lepr in Lepr^neg^Mmp^high^ fibroblasts are caused by TNFα and TGFβ‐induced Tcf21 downregulation. (A) The ranking of transcription factors with reduced expression prevalence in the Lepr^neg^Mmp^high^ subset compared to the Fb3 subset. (B) Feature plots of expression distribution for *Tcf21* in the GFP^+^ fibroblastscRNA‐seq dataset (Figure S1I). (C) Feature plots of expression distribution for *TCF21* in the human CRC fibroblast scRNA‐seq dataset (Figure 1H). (D) Spearman correlation of *LEPR* and *TCF21* in human colorectal tissues based on GTEx database (p < 0.0001, R = 0.71). (E) qRT‐PCR analysis for *Lepr* and *Csf3* in GFP^+^ fibroblasts treated with siNC or siTcf21 for 48 h (n = 3). (F) Transwell assay showing migration of macrophages induced by the supernatants from GFP^+^ fibroblasts with siNC or siTcf21 treatment (n = 3). (G) Quantification of cell migration in panel F. (H) Schematic diagram showing two potential TCF21 binding sites at Lepr promoter. (I) Luciferase activity in lysates of HEK293T cells transfected with luciferase reporter plasmids of pGL3‐basic empty vector (pGL3‐basic), wild‐type LEPR promoter (pLepr‐WT), or LEPR promoter with mutations (pLepr‐Mut1, pLepr‐Mut2) in TCF21 binding sites under normal and Tcf21 overexpression conditions (n = 4). (J) CUT&RUN‐qPCR analysis of Tcf21 occupancy at two predicted binding sites within the *Lepr* promoter. (K) qRT‐PCR analysis of *Tcf21* in GFP^+^ fibroblasts treated with TNFα and/or TGFβ for 36 h (n = 3). (L) Representative gross images of AOM‐DSS colon tumors from control (*Tcf21^con^*) and *Pdgfrα* ^CreERT^; *Tcf21* ^fl/fl^ (*Tcf21^cko^*) mice. n = 5 mice for each group. (M) Quantification of the number of tumors from *Tcf21^con^* and *Tcf21^cko^* mice in panel L. (N) Quantification of the diameter of tumors from *Tcf21^con^* and *Tcf21^cko^* mice in panel L. *Tcf21^con^*: n = 49 tumors from 5 mice; *Tcf21^cko^*: n = 90 tumors from 5 mice. (O) Representative histological images of AOM‐DSS colon tumors from *Tcf21^con^* and *Tcf21^cko^* mice in panel L. Scale bar, 2 mm. (P) Representative Immunofluorescence images of CD45 and F4/80 in colon tumors from mice in panel L. n = 4 mice for each group. Scale bar, 50 µm. (Q) Quantification of the percentage of F4/80^+^ cells in panel P. Data are presented as mean ± SEM (E,G,I–K,M–Q). Unpaired t test (E,G,J,K,M,Q) and Mann–Whitney test (N) were used. Brown‐Forsythe and Welch ANOVA test (I) were used. ^**^ *p* < 0.01, and ^***^ *p* < 0.001. See also Figure S9.

To assess the clinical relevance of Tcf21 in CRC, we analyzed its expression in human colon tissues from TCGA database, finding that Tcf21 mRNA levels were significantly lower in tumor tissues (Figure S9H). Simultaneously, combined TNFα and TGFβ treatment leads to a significant downregulation of *Tcf21* expression in *Lepr* ^+^ fibroblasts (Figure 6K). To investigate the in vivo role of Tcf21 in CRC progression, we generated *Pdgfra^CreERT2^;Tcf21^fl/fl^* (*Tcf21^cko^*) mice, in which exon 1&2 of *Tcf21* was specifically deleted in fibroblast (Figure S9I–J). Notably, *Tcf21* deletion significantly reduced the number and size of colonic adenomas (Figure 6L–O). In addition, the loss of *Tcf21* led to a marked increase in F4/80^+^ macrophages number, mirroring the phenotype observed in *Lepr*‐deficient mice (Figure 6P,Q). Taken together, these data indicate that combined TNFα and TGFβ signaling induce *Tcf21* deficiency, which downregulates *Lepr* in Lepr^neg^Mmp^high^ cells that transition from Lepr‐lineage cells, thereby promoting CRC progression via enhanced macrophage infiltration.

## 3 Discussion

CAFs are key regulators of CRC progression, in part through their ability to shape the immune microenvironment. In particular, inflammatory fibroblast states that promote myeloid cell recruitment have emerged as critical drivers of tumor development [32, 33]. However, how these tumor‐promoting fibroblast states arise and are regulated within the TME remains poorly understood. In this study, we identify a tumor‐associated fibroblast state characterized by enhanced inflammatory and macrophage‐recruiting capacity, which is selectively enriched in CRC. We show that this state arises from resident fibroblasts in response to tumor‐derived inflammatory cues, rather than pre‐existing lineage. Mechanistically, this transition is associated with destabilization of a Tcf21‐Lepr‐dependent regulatory program, which normally constrains fibroblast activation. Together, our findings define a tumor‐driven fibroblast state transition that links inflammatory signaling to the emergence of a macrophage‐recruiting, tumor‐promoting stromal niche.

Inflammatory and fibrotic cytokines within the TME are major determinants of fibroblast behavior [34]. Previous studies have reported the effects of individual cytokines in driving fibroblast activation or differentiation in tumors [35, 36]. However, our study indicates that the Lepr^neg^Mmp^high^ iCAF state, characterized by elevated inflammatory mediator expression, is driven by the combined TNFα and TGFβ signaling, which elicit divergent downstream transcriptional programs. Notably, although both TNFα and TGFβ individually downregulate Lepr expression, knockdown of Lepr alone is insufficient to recapitulate this phenotype. Instead, Lepr loss contributes to specific functional features of this state, including increased Csf3 production and enhanced macrophage‐recruiting capacity. These findings suggest that Lepr^neg^Mmp^high^ fibroblast state cannot be attributed to Lepr loss alone, but instead reflects the integration of inflammatory signaling cues. This model is consistent with recent work showing that cooperative inflammatory signal, such as TNFα and IL‐1, drives the emergence of distinct IL11^+^ iCAF subsets in head and neck cancer [37]. Previous studies have demonstrated that TGFβ alone promotes myofibroblastic differentiation of Lepr^+^ fibroblasts in CRC [38]. In contrast, our findings reveal that the addition of TNFα signaling in the context of TGFβ reshapes the trajectory of Lepr^+^ fibroblast state transitions, biasing them toward inflammatory and immunomodulatory phenotypes. These results thus extend prior work by highlighting how combinatorial signaling inputs redefine fibroblast cell states.

Our data further implicate the Tcf21‐Lepr axis as an intrinsic regulatory module associated with fibroblast identity and quiescence. Tcf21 is widely recognized as a transcription factor enriched in resting fibroblasts across multiple mesenchymal tissues, where it contributes to maintenance of a non‐activated cellular state [39]. In CRC, we observe coordinated downregulation of Tcf21 and Lepr in fibroblasts exposed to tumor‐associated inflammatory signals, suggesting that repression of this axis accompanies loss of fibroblast homeostatic features. Functional perturbation of Tcf21 in fibroblasts phenocopies key aspects of Lepr loss, including enhanced inflammatory gene expression and increased capacity to recruit macrophages, supporting a model in which Tcf21 functions upstream to stabilize Lepr expression. These findings position Tcf21 as a transcriptional regulator of Lepr, and to our knowledge, this represents the first reports identifying a direct regulatory axis between these two factors. Supporting this notion, prior studies have shown that Tcf21 and Lepr play crucial roles in maintaining the quiescent or progenitor‐like state of stromal cells in multiple organs, including the heart [40], kidney [41], bone marrow [42], and visceral adipose tissue [43]. Loss of either gene in these contexts consistently results in the differentiation of stromal cells into more specialized or pro‐inflammatory phenotypes, often accompanied by extracellular matrix remodeling and immune cell infiltration. These findings support the view that this regulatory module delineates a homeostatic fibroblast state that is progressively dismantled during tumor development. Loss of Tcf21‐Lepr expression therefore reflects a transition away from a restrained stromal program toward a functionally reprogrammed state with enhanced inflammatory and immunomodulatory output in the CRC microenvironment.

Functionally, our findings highlight Csf3 as a notable transcriptional output of the Lepr^neg^Mmp^high^ fibroblast state. Csf3 (G‐CSF) is produced by multiple cell types, including activated T cells, monocytes, and macrophages under inflammatory conditions [44, 45], which is classically associated with granulocyte mobilization and myeloid responses [46, 47]. Previous studies have demonstrated that CSF3 is enriched in CRC and is associated with poor prognosis [27]. However, the precise cellular source of CSF3 remains to be defined. In our study, its expression is highly restricted to the Lepr^neg^Mmp^high^ and hFb4 fibroblast subset, rather than immune or epithelial cell type in both mouse and human CRC. This selective enrichment suggests that Csf3 represents a state‐associated feature of reprogrammed fibroblasts rather than a general marker of tumor‐associated inflammation. We demonstrate that Csf3 is required for the macrophage‐recruiting phenotype associated with fibroblast reprogramming, thereby establishing a functional link between fibroblast state transition and myeloid cell accumulation. Together, these results support a model in which inflammatory reprogramming of fibroblasts gives rise to a specialized stromal state that contributes to shaping the tumor immune microenvironment through selective production of myeloid‐regulating factors.

In summary, we identified a tumor‐driven fibroblast state transition in which homeostatic Lepr^+^ fibroblasts lose their identity and acquire inflammatory and immune‐modulatory functions during CRC progression. This transition is driven by the integration of TNFα and TGFβ signaling within the TME and is associated with destabilization of a Tcf21‐Lepr regulatory program. Functionally, this transition enables fibroblasts to remodel the tumor immune microenvironment via state‐restricted production of CSF3, thereby promoting macrophage accumulation and tumor development. These results highlight inflammatory signaling and fibroblast identity programs as potential points of therapeutic intervention to modulate stromal‐immune interactions in CRC.

## 4 Limitations of the Study

While our findings provide evidence for fibroblast‐to‐iCAF trajectory during CRC progression, certain questions remain unresolved. First, although we demonstrate that TNFα and TGFβ cooperatively downregulate *Tcf21*, the precise molecular mechanism underlying this regulation remains to be elucidated. It is plausible that Smad2/3 (downstream of TGFβ) and NF‐κB (downstream of TNFα) directly repress the *Tcf21* promoter or modulate its chromatin accessibility, as suggested by previous studies [48, 49]. Future investigations employing chromatin immunoprecipitation and ATAC‐seq will be instrumental in determining whether *Tcf21* silencing is mediated through direct transcriptional repression or broader epigenetic remodeling. Second, despite the robust induction of Csf3 following Lepr loss, defining the downstream signaling remains challenging. In particular, pharmacological inhibition of NF‐κB and STAT3 signaling did not attenuate Csf3 upregulation, indicating that Lepr‐dependent regulation of Csf3 is unlikely to be mediated through these canonical inflammatory pathways (Data not shown). These results highlight the complexity of Lepr signaling in fibroblasts and suggest that raise the possibility that Csf3 induction may occur through indirect or previously unrecognized mechanisms. Accordingly, unbiased approaches will be necessary to identify the molecular regulators linking Lepr deficiency to Csf3 induction. Third, although transwell assays support a direct effect of fibroblast‐derived factors on macrophage migration, and scRNA‐sequencing analysis identified an inflammation‐associated macrophage state enriched in hFb4‐high tumors, these findings do not establish a direct in vivo causal link between fibroblasts and macrophage accumulation in tumors. Increased macrophage abundance may also reflect local proliferation, prolonged survival, or shifts between resident and monocyte‐derived macrophage populations. Moreover, GFP^+^ donor fibroblasts were no longer reliably detectable in endpoint tumors approximately 1 month after co‐transplantation, precluding robust assessment of their persistence and its relationship with macrophage accumulation at this late time point. Further lineage‐tracing and longitudinal studies will be required to further define the persistence and temporal dynamics of fibroblast‐macrophage interactions in vivo.

## 5 Experimental Model and Study Participant Details

### 5.1 Animals

All animal experimental procedures and protocols were authorized by Beijing Laboratory Animal Management and were performed in strict accordance with the guidelines of the Institutional Animal Care and Use Committee of China Agricultural University (approval number: AW81212202‐3‐1). *Lepr‐Cre* (JAX stock number: 008320, RRID: IMSR_JAX:008320), *Rosa26‐mTmG* (JAX stock number: 007676, RRID: IMSR_JAX:007676) and *Lepr* ^flox/flox^ (JAX stock number: 008327, RRID: IMSR_JAX:008327) mice were obtained from Jackson Laboratories. *Tcf21* ^flox/flox^ mice (Strain NO. T013083, RRID: IMSR_GPT: T013083) were purchased from GemPharmatech (Nanjing, China). *Pdgfra‐Cre^ERT2^* mice were provided by Hua Zhang's laboratory at China Agricultural University. C57BL/6JNifdc mice were obtained from Charles River Laboratory.

### 5.2 Patients and Clinical Specimens

Clinical human CRC tissues were collected from the First Affiliated Hospital of Zhengzhou University. Tumor tissues were fixed in 4% paraformaldehyde in PBS overnight, dehydrated in gradients. subsequently embedded in paraffin. Thin slices with a thickness of 4 µm were prepared for immunostaining analyses. All human specimens included in this study were collected with informed consent and approved by the Institutional Review Board and the Ethics Committee of Zhengzhou University (nr: 2021‐KY‐0121‐001).

### 5.3 AOM and DSS‐induced Colon Tumor Mouse Model

Two‐month‐old mice were intraperitoneally injected with AOM (Sigma‐Aldrich, Cat# A5486) at a dose of 10 mg/kg body weight. Five days after AOM injection, mice were subjected to three cycles of dextran sulfate sodium (DSS) treatment. Each cycle comprised 5 days of 2% (w/v) DSS (MP Biomedicals, Cat# 218397125, molecular weight: 36 000—50 000) administration in drinking water, followed by 14 days of normal water. After treatment, mice were sacrificed, and distal colon tissues were collected for analysis.

## 6 Method Details

### 6.1 Histology, Immunofluorescence, and Immunohistochemistry

For histological analysis, colon tissues were fixed in 10% zinc formalin at room temperature for 24 h, rinsed with 1× DPBS, then embedded in paraffin, and sectioned at 5 µm. Sections were deparaffinized with xylene and rehydrated through gradient ethanol. Sections were stained with hematoxylin (Sigma‐Aldrich, 03971) for 6 min, washed in tap water for 5 min, and counterstained with eosin (Sigma‐Aldrich, 318906) for 5 sec. Sections were subsequently dehydrated with gradient ethanol and sealed finally.

For immunohistochemistry, sections were deparaffinized with xylene and rehydrated through a graded ethanol series. Antigen retrieval was performed using either Tris/EDTA buffer (pH 9) or sodium citrate buffer (10 mM, pH 6) in a microwave for 20 min. After cooling to room temperature, sections were incubated with 3% hydrogen peroxide for 20 min to block endogenous peroxidase activity and then blocked with a blocking agent for 1 h. Sections were incubated overnight at 4°C with primary antibodies. Immunostaining was conducted using the ABC peroxidase method (Vector Laboratories, PK‐6101) with diaminobenzidine as the substrate and hematoxylin as the counterstain. Slides were sealed with neutral gum.

For immunofluorescence staining, after antigen retrieval and blocking, sections were incubated overnight with primary antibodies. Secondary labeling was performed using secondary antibodies for 1 h at room temperature, followed by nuclear staining with DAPI (Beyotime, C1002) for 20 min. Sections were then covered with an anti‐quenching reagent.

The following antibodies were used: GFP (Abcam, ab13970, RRID: AB_300798, 1:800), Il11 (Thermo Fisher, PA5‐36544, RRID: AB_2553579, 1:100), MMR (R&D system, AF2535, RRID: AB_2063012, 1:50), ST2(Proteintech, 11920‐1‐AP, RRID: AB_906359, 1:400), MMP3 (Proteintech, 66338‐1‐lg, RRID: AB_2881718, 1:200), CD45 (CST, 70257, RRID: AB_2799780, 1:200), F4/80 (CST, 70076, RRID: AB_2799771, 1:400), Goat anti‐Rabbit IgG (H+L) Cross‐Adsorbed Secondary Antibody, Alexa Fluor 594, (Thermo Fisher, Cat# A‐11012, RRID: AB_2534079, 1:400), Goat anti‐Mouse IgG (H+L) Highly Cross‐Adsorbed Secondary Antibody, Alexa Fluor 488 (Thermo Fisher, Cat# A‐11029, RRID: AB_2534088, 1:400), Goat anti‐Mouse IgG (H+L) Highly Cross‐Adsorbed Secondary Antibody, Alexa Fluor 647 (Thermo Fisher, Cat# A‐21236, RRID: AB_2535805, 1:400), Goat anti‐Chicken IgY (H+L) Secondary Antibody, Alexa Fluor 488 (Thermo Fisher, Cat# A‐11039, RRID: AB_2534096, 1:400).

### 6.2 Frozen Section

Following embedding in OCT (Tissue‐Tek; Sakura Finetek) and freezing on dry ice, tissues were cryosectioned into 4 µm sections. These sections underwent air‐drying (30 min), three PBS washes, and DAPI staining. Images were acquired with a Leica TCS SP8 laser scanning confocal microscope. Image were captured by a microscope (LEICA DM6 B) with LEICA DFC9000 GT camera.

### 6.3 QRT‐PCR

Total RNA was extracted from sorted cells of cell lines and mouse colonic tissues using TRIzol reagent (Thermo Fisher, 15596018CN) according to the manufacturer's instructions. For mRNA detection, reverse transcription was performed using oligo(dT) primers. qRT‐PCR was carried out using ChamQ Universal SYBR qPCR Master Mix (Vazyme, Q711‐02) on a LightCycler 480 Real‐Time PCR System (Roche, Mannheim). Relative expression levels were calculated using the 2^ΔΔ^CT method, with GAPDH serving as the internal control. The primers used for gene assessment were as follows:

Lepr‐forward GCATCCCTGCTTTACGCCAC
Lepr‐reverse GGAGAAGTTGCGTGGCTCT
GAPDH‐forward AGGTCGGTGTGAACGGATTTG
GAPDH‐reverse TGTAGACCATGTAGTTGAGGTCA
Il11‐forward TGTTCTCCTAACCCGATCCCT
Il11‐reverse CAGGAAGCTGCAAAGATCCCA
Il1rl1‐forward CGGAGAATGGAACCAACTACATCGT
Il1rl1‐reverse GCCACAGGACATCAGCCAAGAAG
Mmp13‐forward CCACAGTTGACAGGCTCCGAGAA
Mmp13‐reverse CCACATCAGGCACTCCACATCTTG
Mmp10‐forward GCCTACCAATCTGCTCAGCGTATC
Mmp10‐reverse TGAAGCCACCAACATCAGGAACAC
Inhba‐forward GTCCTCGCTCTCCTTCCACTCAA
Inhba‐reverse AGCAGCCACACTCCTCCACAAT
Mmp9‐forward CGCCACCACAGCCAACTATGAC
Mmp9‐reverse TACTGGATGCCGTCTATGTCGTCTT
Mmp3‐forward GCCAGGATTTCCCAGGAAGATAGC
Mmp3‐reverse CCAACTGCGAAGATCCACTGAAGAA
Tcf21‐forward GCTCTCCAAGCTGGACACTC
Tcf21‐reverse GTTGGCCAGGATCTGCCTTA
Csf3‐forward GCACTATGGTCAGGACGAGAGG
Csf3‐reverse GCTGGCTTAGGCACTGTGTCT

### 6.4 Tamoxifen, Inhibitors and Clodronate Liposomes Treatment

To knockout *Lepr* or *Tcf21* in fibroblasts, the mice were intraperitoneally injected with tamoxifen (Sigma‐Aldrich, T5648) diluted in corn oil (Sigma‐Aldrich, C8267) at a dose of 4 mg pre 25 g body weight after induction with AOM/DSS. Littermate control and conditional knockout mice were used in all experiments to ensure comparable genetic background.

For R‐7050 (TNFα inhibitor, MCE, HY‐110203) and LY2109761 (TGFβ inhibitor, MCE, HY‐12075) treatments, the AOM‐DSS‐induced colorectal tumor model was established using *Lepr‐Cre;mTmG* mice. Littermate mice were randomly assigned to control and treatment groups. TNFα and TGFβ inhibitors were prepared according to the manufacturer's instructions and administered once every 3 days, starting after the third round of DSS induction. R7050 was administered intraperitoneally at a dose of 6 mg/kg, and LY2109761 was administered orally at a dose of 12 mg/kg. Littermates were randomly assigned to different experimental groups.

For clodronate liposome (Yeasen, 40337ES10) treatment, the AOM‐DSS‐induced colorectal tumor model was established using *Lepr^con^* and *Lepr^cko^* mice. Each of mice was intraperitoneally injected with 200 µL of clodronate liposomes every 3 days, beginning after the third round of DSS induction. Littermate control and conditional knockout mice were used in all experiments to ensure comparable genetic background.

### 6.5 Orthotopic Co‐Implantation of Tumor and Fibroblasts in Cecum

Six‐week‐old male C57BL/6J mice were randomly assigned to experimental groups (n = 12 per group). Mice were anesthetized with Zoletil50 (Virbac), and upon loss of the righting reflex, the cecum was exteriorized via laparotomy. Once the righting reflex was lost, the cecum was exteriorized via laparotomy. GFP^+^ fibroblasts or induced GFP^+^ fibroblasts (3 × 10^6^) were co‐injected orthotopically with MC38 cells (1 × 10^6^) into the subserosal layer of the cecum. After injection, the gut was returned to the abdominal cavity and the incision closed. To prevent postoperative infection, mice were subcutaneously injected with Baytril (Bayer) for three consecutive days. Tumors were harvested at 1 month after injection.

### 6.6 Cell Culture and Transfections

HEK293T (RRID: CVCL_B288) and MC38 (RRID: CVCL_0063) cell lines were purchased from the National Biomedical Cell‐Line Resource (NSTI‐BMCR) (Beijing, China) and maintained in DMEM (Cytiva, SH30243.01) supplemented with 10% fetal bovine serum (FBS) (Thermo Fisher, 10091148) and 1% Penicillin‐Streptomycin (Thermo Fisher, 15140148). All cell lines were tested and confirmed to be free of mycoplasma contamination. Cells were maintained and used within 15 passages to ensure genetic stability and experimental reproducibility. Transfections were performed using Lipofectamine RNAiMAX reagent (Thermo Fisher, 13778150) according to the manufacturer's protocol, with 2 µg of vector or negative control vector per well in a 6‐well plate. Samples were collected at 24 or 48 h after transfection.

### 6.7 Flow Cytometry and Cell Sorting

GFP^+^ cells and CD45^+^CD11b^+^F4/80^+^ cells were sorted by flow cytometry. Single‐cell suspensions from *Lepr‐Cre;mTmG* mice were stained with Fixable Viability Dye (eBioscience, 65‐0863‐14) for 15 min to exclude dead cells. Single‐cell suspensions from *Lepr^con^* and *Lepr^cko^* mice were stained using Calcein‐AM (Biolegend, 425201, 1:100), CD45‐APC (eBioscience, 170451–82, RRID: AB_469392, 1:500), CD11b‐BV711 (Biolegend, 101241, RRID: AB_11218791, 1:400), F4/80‐BV421 (Biolegend, 123131, RRID: AB_10901171, 1:400).

### 6.8 Isolation and Culture of Fibroblasts

#### 6.8.1 GFP^+^ Colon Fibroblast Isolation

Colon fibroblasts were isolated with minor modifications as previously described. Briefly, colons were placed in cold HBSS to remove debris. The tissues were then opened longitudinally and incubated in PBS containing 20 mM EDTA for 20 min at 37°C to detach primary colon epithelial cells. After vigorous shaking to remove the epithelial layer, the remaining tissue was rinsed several times with cold modified DMEM/F12 and subsequently incubated in a digestion solution containing 300 U/mL collagenase II (Thermo Fisher, 17101015) and 0.05 U/mL dispase (STEMCELL, 07913) for 50 min at 37°C on a rotating platform. Following vigorous shaking, single cells were pelleted by centrifugation at 350 × *g* for 5 min. The supernatant was discarded, and the cells were resuspended in fresh medium. GFP^+^ fibroblasts were isolated from the suspension by flow cytometry for droplet‐based 3ʹ‐scRNA‐seq analysis (10× Genomics).

#### 6.8.2 GFP^+^ Cancer Associated Fibroblast Isolation

Tumors were dissected from the colon, washed with cold HBSS, and minced into small pieces. The tissue was incubated in a digestion solution containing 600 U/mL collagenase II (Thermo Fisher, 17101015), 0.05 U/mL DNase I (Merck, DN25), and 0.05 U/mL dispase for 30 min at 37°C on a rotating platform. After vigorous shaking, single cells were pelleted by centrifugation at 350 × *g* for 5 min. The supernatant was discarded, and the cells were resuspended in fresh medium. GFP^+^ cancer‐associated fibroblasts were isolated by flow cytometry for droplet‐based 3ʹ‐scRNA‐seq analysis (10× Genomics).

#### 6.8.3 Fibroblast Culture

After tissue digestion and centrifugation, the supernatant was discarded, and each pellet was resuspended in modified DMEM (Thermo Fisher, 10569010) supplemented with 10% FBS, 1% Penicillin‐Streptomycin, and 1× nonessential amino acids (NEAA, Thermo Fisher, 11140050). Cells were seeded in a 6‐cm dish and cultured in a humidified incubator at 37°C with 5% CO_2_. After 2 h, floating debris and nonadherent cells were removed by washing with PBS. Adherent cells, identified as fibroblasts, were maintained under these conditions. Cells were passaged at a 1:2 ratio 5 days after seeding. GFP^+^ fibroblasts were isolated by flow cytometry after three passages for subsequent experiments.

#### 6.8.4 Cytokine Treatment

GFP^+^ fibroblasts were seeded in plates and cultured for 12 h prior to treatment with the respective cytokines, followed by an additional 36‐h incubation period under standard culture conditions. The following cytokines were used: TNFα (20 ng/mL, Peprotech, 315‐01A), TGFβ (10 ng/mL, Sinobio, 80116‐RNAH), Areg (20 ng/mL, MCE, HY‐P77868), Hbegf (20 ng/mL, MCE, HY‐P7194), TGFα (20 ng/mL, MCE, HY‐P77857).

### 6.9 Bone Marrow‐Derived Macrophage (BMDM) Culture and Transwell Migration Assay

BMDMs were prepared as described previously [50]. Bone marrow cells were isolated from the tibiae and femora of 8‐10‐week‐old C57BL/6J mice under sterile conditions. After removal of the epiphyses, marrow was flushed out, dispersed into a single‐cell suspension, and passed through a 70‐µm cell strainer. Cells were pelleted by centrifugation at 500 × *g* for 5 min and resuspended in macrophage complete medium consisting of DMEM supplemented with 10% (FBS), 1% Penicillin‐Streptomycin, and 20 ng/mL macrophage colony‐stimulating factor (M‐CSF, 315‐02, Peprotech). Cells were plated in 6‐cm dishes and maintained at 37°C with 5% CO_2_. On day 4, an additional 5 mL of complete medium was added, and on day 7, differentiated BMDMs were harvested for Transwell assays.

Conditioned media (CM) were collected from GFP^+^ fibroblasts treated with vehicle (Veh) or cytokines (TNFα and TGFβ) and placed in the lower chambers of Transwell plates (#3422, Corning) as chemoattractants. BMDMs were seeded in the upper chambers and allowed to migrate for 3 h. Cells that migrated to the lower surface of the membrane were fixed, stained with crystal violet, imaged under a bright‐field microscope, and quantified.

### 6.10 Luciferase Assays

The full‐length mouse Tcf21 construct was cloned into a pcDNA3.1 vector. The Lepr gene is located on chromosome 4 (NC_000070.7, base pairs 101574393–101676375) in the mouse genome. An approximately 2‐kb region upstream of the transcript start site (TSS) was identified as the Lepr promoter (chromosome 4, NC_000070.7: base pairs 101572393–101574393) and cloned into the pGL3‐Basic reporter vector. Tcf21 binding sites 1 and 2 are located at base pairs 101574271–101574285 and 101572863–101572877, respectively. The construct was verified by DNA sequencing. Site‐directed mutagenesis (BGI, Shenzhen, China) was used to mutate nucleotides at the Tcf21 binding sites. Wild‐type and mutant plasmids were co‐transfected with the phRL‐TK control plasmid into HEK293T cells. Luciferase activities of firefly and Renilla were measured 24 h post‐transfection using the Dual‐Glo Luciferase Assay Kit (Promega, E2980) according to the manufacturer's instructions.

### 6.11 Single Cell Library Generation and Processing of scRNA‐seq Data

GFP^+^ fibroblast single‐cell suspensions from tumor and normal samples were prepared as described above, with three mice included per sample. The number of viable cells in 10 µL of each cell suspension was counted using an automated cell counter (Luna‐II, Logos Biosystems). Cell viability and concentration were then assessed using the Rigel S3 fluorescence cell analyser (Countstar). Following the manufacturer's instructions, cells were loaded into a Chromium Single‐Cell Controller (10x Genomics) to generate single‐cell gel beads in emulsion. The scRNA‐seq library was constructed using the Single Cell 3′ Library and Gel Bead Kit v2 and the sequencing was performed on a NovaSeq 6000 sequencer (Illumina).

Raw FASTQ reads from scRNA‐seq were mapped and quantified using CellRanger toolkit v6.1.2 (RRID: SCR_017344) by aligning to the mouse mm10 reference genome. Cells that met the following three criteria were retained and used for subsequent analysis: (1) the total UMI count per cell was less than 8,000; (2) the number of detected genes were less than 2,500; (3) the proportion of mitochondrial genes was below 10%.

### 6.12 Clustering Analysis of scRNA‐seq Data

Clustering analysis of scRNA‐seq data was performed using the Seurat [51] (version 3.4) and harmony [52] (version 0.1.1) R packages. For mouse tumor and normal samples, we subset out the GFP^+^ cells for further analysis. Briefly, single cell data matrices from normal and tumor samples were merged, library size‐normalized, log‐transformed and then integrated using the RunHarmony function by following the standard workflow described in harmony package vignettes. To identify cell clusters, 3,000 highly variable genes (HVGs) were identified, and the top 40 principal components (PCs) were used as an input of the RunHarmony function. Then the corrected Harmony embeddings were used to find cell clusters with a resolution = 0.05. The identified cell clusters were merged into major cell types based on the expression of well‐known marker genes. FindAllMarker function was used to find marker genes for each cluster by following the parameters: test.use = “wilcox”, logfc.threshold = 0.25 and min.pct = 0.25. For subclustering analysis of fibroblasts, we subset out the fibroblast cell type and then perform the integration analysis. The top 30 PCs with a resolution = 1.0 were used to find fibroblast subclusters, which were then merged based on the expression patterns of marker genes.

### 6.13 Analysis of Human Fibroblasts and Macrophages scRNA‐seq Data

We re‐analyzed human fibroblasts scRNA‐seq data with 45,581 cells from a previously published human CRC atlas, which comprised 427 human colon and rectum samples from 192 donors divided into six groups, including healthy, uninflamed, inflamed, polyp, paracancerous and tumor samples [19]. Clustering analysis of these human fibroblasts excluding the two clusters annotated as pericytes and smooth muscle was performed using the harmony package as described above. Eight fibroblast clusters (0, 1, …, 7) were obtained with the top 40 PCs and a clustering resolution of 0.15. Given that the majority of cluster 1 cells were from tumor samples, we further performed subclustering analysis of cluster 1, leading to eight subclusters (1‐0, 1‐1, …, 1–7) with the top 20 PCs and a clustering resolution of 0.3. An appropriate clustering resolution was determined based on the specificity of the identified marker genes in each cluster from differential expression analysis. We finally obtained 13 fibroblast subclusters (hFb1, hFb2, …, hFb13).

Similarly, 42,296 macrophage/monocyte (Macro/Mono) cells from the CRC Atlas were re‐analyzed using the same parameters and analytical workflow as those used for fibroblast subpopulation analysis. CD68^+^CD106^+^ cells were retained for downstream analyses, leading to the identification of eight macrophage subpopulations (Macro_C1–Macro_C8).

For each sample, the relative abundance of hFb4 cells in normal and CRC tissue was calculated as the proportion of hFb4 cells among all fibroblasts in each sample. Samples were then stratified into hFb4‐high and hFb4‐low groups according to the median hFb4 abundance. The relative abundance of each macrophage subpopulation among total macrophages was compared between the two groups using the Wilcoxon rank‐sum test. In parallel, Spearman's rank correlation analysis was performed across samples to evaluate the association between the relative abundance of hFb4 cells among total fibroblasts and that of each macrophage subpopulation among total macrophages.

### 6.14 Analysis of Mouse CRC scRNA‐seq Data

We reanalyzed a mouse colorectal cancer single‐cell RNA sequencing dataset comprising 11,259 cells [18]. Cells from the Adj_normal, AOM/DSS, and WT groups were retained for downstream analysis, with the WT and Adj_normal groups collectively designated as the Normal group. Data integration was performed as described previously. Following integration, cells were clustered at a resolution of 1.0, and the resulting clusters were annotated and consolidated into major cell types based on the expression of established canonical marker genes, including B cells, CD4^+^ T cells, CD8^+^ T cells, endothelial cells, epithelial cells, erythroid cells, fibroblasts, macrophages, mast cells, neutrophils, plasma cells, and proliferating T cells.

Fibroblasts were subsequently extracted for further subclustering. Following data integration, fibroblast subpopulations were identified using the first 30 principal components (PCs) at a clustering resolution of 1.3. The resulting clusters were annotated and consolidated based on their marker gene expression profiles. Pericytes were excluded from the fibroblast compartment, yielding five distinct fibroblast subpopulations for downstream analyses.

### 6.15 Gene Set Scoring and Pathway Enrichment Analysis

Gene set scoring was performed on single cells of normal and tumor fibroblasts from both murine and human sources using the AUCell function from Seurat package. The functional signatures for wound healing (GO:0042060), glycolysis (Hallmark database), inflammatory response (GO: 0006954), and degradation of ECM (Reactome database) were downloaded from the MSigDB web (https://www.gsea‐msigdb.org/gsea/msigdb/). The marker genes of the Lepr^neg^Mmp^high^ fibroblast subpopulation in mouse‐derived tumor fibroblasts were identified using *FindAllMarkers* with the parameters described above. The top 30 marker genes were used for gene scoring analysis. The signaling pathway activities of mouse‐derived fibroblast subpopulations were inferred using the PROGENy R package [23].

### 6.16 Cell–Cell Communication Analysis

The cell‐cell communication analysis of human scRNA‐seq data was conducted by following the tutorial of CellChat R package [53, 54]. To infer cell‐cell communication between fibroblasts and other cells within TME, such as immune and endothelial cells, we combined the identified 13 fibroblast subclusters (hFb1, hFb2, …, hFb13), the 12 major immune cell types (B: B cells, Plasma, DC: dendritic cells, Mono: monocytes, Macro: macrophages, MAST: mast cells, Prolif. MYL: proliferative myeloid cells, CD4: CD4^+^ T cells, CD8: CD8^+^ T cells, Prolif. T: proliferative T cells, NK: NK cells, ILC: innate lymphoid cells), the epithelial cells (Epi: epithelial/malignant cells) and other stromal cells (Endo: endothelial cells, Glial: glial cells) from the previous human CRC atlas, leading to a total of 28 cell groups for cell‐cell communication analysis. To infer cell‐cell communication between interacting cell groups, we computed the average expression of signaling molecules per cell group using 10% truncated mean, and reported the significant cell–cell communication with p‐values < 0.05. Scatter plots were applied to visualize the outgoing and incoming interaction strength of each cell group, and circle plots were used to illustrate the inferred cell‐cell communication.

Cell‐cell communication analysis of the mouse CRC atlas was conducted using the same analytical framework as that applied to the human CRC single‐cell RNA‐seq dataset. Genes expressed in more than 25% of cells were considered highly expressed and were retained for downstream analysis. Communication strength was estimated using the “truncatedMean” method without applying an additional truncation threshold.

### 6.17 Spatial Transcriptomic and Subtype‐Niche Association Analysis

The CRC Xenium formalin‐fixed paraffin‐embedded (FFPE) dataset was integrated using the methods and parameters described in previous study [55]. Cell types were annotated based on the expression of established canonical markers, resulting in the identification of major cell populations, including epithelial cells, T cells, fibroblasts, myeloid cells, endothelial cells, plasma cells, vascular smooth muscle cells, B cells, and mast cells. Fibroblasts and myeloid cells were subsequently extracted for further subclustering. Fibroblasts were classified into six subtypes (ST‐Fib1, ST‐Fib2, …, ST‐Fib6), whereas myeloid cells were further classified into cDC1 cells, monocytes, and macrophages based on the expression of canonical lineage‐specific markers. The annotated cell subtypes were then mapped back to their original spatial coordinates to characterize the spatial distribution of ST‐Fib4 and macrophages.

For the initial spatial niche analysis, the cell‐type composition of the 30 nearest neighbors of each cell was determined. Cells were then clustered based on their local neighborhood composition using the kmeans function in the R package stats, with nstart = 50 and iter.max = 100, yielding nine spatial niches. For each sample within each niche, the observed‐to‐expected (O/E) abundance ratio of each cell subtype was calculated relative to its expected background abundance. Within each niche, Spearman's rank correlation analysis was performed across samples to assess the association between the O/E ratios of ST‐Fib4 and macrophages. Niches were further stratified into ST‐Fib4‐high and ST‐Fib4‐low groups according to the median ST‐Fib4 O/E ratio, and macrophage O/E ratios were compared between the two groups using the Wilcoxon rank‐sum test.

To further characterize macrophage heterogeneity, macrophages were extracted and subclustered using the same analytical approach applied to fibroblast subtyping, resulting in nine macrophage subtypes (ST‐Macro_C1 to ST‐Macro_C9). To assess their transcriptional correspondence with macrophage populations identified by the human CRC scRNA‐seq dataset, marker genes of the Macro_C3 subtype were examined across the Xenium‐derived macrophage subtypes. The expression patterns of these markers supported the correspondence between Macro_C3 and ST‐Macro_C3.

To investigate the spatial proximity between ST‐Fib4 and macrophage subtypes, the Euclidean distance from each macrophage cell to its nearest ST‐Fib4 cell was calculated using the original Xenium spatial coordinates. The nearest‐neighbor distance distributions of different macrophage subtypes were visualized using kernel density estimation separately for each sample. To evaluate the significance of the spatial proximity between ST‐Macro_C3 and ST‐Fib4, the proportion of ST‐Macro_C3 cells located within 50 µm of their nearest ST‐Fib4 cell was calculated. A permutation test was performed independently for each sample using 9,999 random permutations of macrophage subtype labels while preserving the observed cell coordinates and the number of cells in each macrophage subtype. The proportion of ST‐Macro_C3 cells within 50 µm of ST‐Fib4 was recalculated for each permutation to generate a null distribution. A one‐sided empirical *p* value was calculated as (1 + the number of permuted statistics greater than or equal to the observed statistic)/(9,999 + 1).

Following macrophage subtype annotation, spatial niches were redefined using the updated cell subtype annotations and the same methods and parameters described above. The O/E abundance ratios of ST‐Fib4 and ST‐Macro_C3 were then calculated for each sample within each newly defined niche. Spearman's rank correlation analysis and the Wilcoxon rank‐sum test were performed to assess the association between ST‐Fib4 and ST‐Macro_C3, following the same procedures previously applied to ST‐Fib4 and macrophages.

### 6.18 Pseudotemporal Trajectory Analysis of Fibroblasts

We performed pseudotemporal ordering of fibroblast cells from the mouse scRNA‐seq data, including four fibroblast subsets, using scEpath package. Briefly, the harmony‐corrected embedding was used as an input for dimension reduction using PHATE package [56]. Based on this reduced PHATE space, scEpath infers lineage relationships between these fibroblast cell states via predicted transition probabilities and reconstructs pseudotime by ordering individual cells along the development path. The calculated pseudotime is rescaled such that it is bounded in [0, 1]. We also identify pseudotime‐dependent genes that are significantly changed over the pseudotime by creating a smoothed version of gene expression using a cubic regression spline [57].

### 6.19 Tumor Matching with the CMS Classification

For the CMS classification, we used the random forest algorithm packaged in CMScaller to predict the probability of each tumor sample belonging to different molecular subtypes [19, 58]. Specifically, the genes in the expression matrix were converted to their corresponding human homologs using HomoloGene. The expression matrix was summarized, log2‐transformed, and then used for CMS classification with CMScaller R package.

### 6.20 RNA‐seq Analysis

Tumor cells were isolated from colon tumors of mice subjected to co‐implantation experiments. Total RNA was isolated from collected colon tumor cells using TRIzol reagent following the manufacturer's instructions. GFP^+^ fibroblasts were treated with vehicle or TNFα&TGFβ and RNA was extracted following standard procedures. RNA samples were sent to Novogene Co., Ltd. for library preparation and sequencing on the Illumina NovaSeq 6000 platform. All data were statistically analyzed online using the NovoMagic data analysis cloud platform (www.magic.novogene.com) or the R software.

GFP^+^ fibroblasts were treated with siNC or siLepr and RNA was extracted following standard procedures. RNA samples were sent to Majorbio BioTech Co., Ltd. for library preparation and sequencing on the Illumina NovaSeq X Plus platform. All data were statistically analyzed online using the Major data analysis cloud platform (https://www.majorbio.com/) or the R software.

### 6.21 In Situ Hybridization

Colon and tumor treated with AOM/DSS were harvested from *Lepr‐Cre;mTmG* mice and fixed in neutral buffered formalin at RT for 24 h before paraffin embedding. The tissues were paraffin‐embedded and sectioned at a thickness of 5 µm. In situ hybridization was performed using RNAscope as per the manufacturer's instructions. An RNAscope Multiplex Fluorescent Reagent Kit v2 (Advanced Cell Diagnostics, 323100) and an RNAscopeTM Probe‐Mm‐Lepr‐C1 (Advanced Cell Diagnostics, 402731‐C1) were used in this experiment.

### 6.22 CUT&RUN‐qPCR Assay

CUT&RUN‐qPCR was performed by Hyperactive pG‐MNase CUT&RUN Assay Kit for PCR/qPCR (Vazyme, HD101). In brief, 5 × 10^5^ GFP^+^ colon fibroblasts from *Lepr‐Cre;mTmG* were suspended and bound with ConA beads for 10 min and then incubated with primary antibodies at 4°C overnight. After washing, pG/MNase Enzyme was added into the sample and incubated for 90 min at 4°C. Additionally, CaCl_2_ was added for DNA fragmentation lasting 90 min on ice and stopped by stop buffer at 37°C for 15 min. To normalize the samples, Spike in was added into stop buffer as uniform correction. Subsequently, the DNA was extracted by FastPure gDNA Mini Columns for qPCR. The following primary antibodies were used: TCF21 Polyclonal Antibody (Thermo Fisher, PA5‐119812, RRID: AB_2913384), Tri‐Methyl‐Histone H3 Rabbit Monoclonal Antibody (CST, #9751, RRID: AB_2616028).

### 6.23 Western Blotting

Total proteins from colon tumor were extracted using a complete lysis buffer (Beyotime, P0013) added PMSF (Beyotime, ST506). For denatured samples, proteins were separated by 10% SDS‐PAGE gels (Epizyme Biotech, PG222) and electro‐transferred to 0.45 µm PVDF membranes (Millipore, IPVH00010) according to the molecular weight of the target proteins, using Prestained Protein Ladder (Thermo Fisher, 26616) as a molecular weight marker. After blocking with 5% skim milk in TBST at room temperature, the membranes were probed with specific antibodies. Protein bands were detected using HRP‐conjugated secondary antibodies (Beyotime, A0208, A0216) and ECL detection reagent (Millipore, WBKLS0500). The following primary antibodies were used: Anti‐G‐CSF antibody (Abcam, ab181053) and GAPDH Mouse Monoclonal Antibody (Beyotime, AF0006).

### 6.24 Cytokine Measurements

Fibroblasts supernatants were centrifuged at 13 000 g and 4°C for 10 min, and the supernatant was collected. The concentrations of cytokines in GFP^+^ fibroblast supernatant were determined according to the instructions of an ELISA kit (Elabscience, E‐EL‐M0031).

### 6.25 Statistical Analysis

At least three mice were included in each experimental group. For cell experiments, each group contained at least three independent biological replicates. All graphs were made using GraphPad Prism software (GraphPad, RRID:SCR_002798). Statistical significance between two groups was determined by paired two‐tailed Student's t test, unpaired two‐tailed Student's t test, or nonparametric t‐test followed by Mann–Whitney test. Statistical significance among three or more groups was determined by one‐way analysis of variance (ANOVA). All data are presented as mean ± standard error of the mean (SEM). p values less than 0.05 were considered statistically significant (^∗^ *p* < 0.05, ^∗∗^ *p* < 0.01, ^∗∗∗^ *p* < 0.001).

## Author Contributions

C.L. and Z.Y. designed the experiments. M.S., B.Q., M.D., L.H., X.Y., J.X., L.Y. and S.T. performed research. S.J. and W.Y. conceptualized and performed computational experiments and analysis. M.S., S.J., W.Y., C.L. and Z.Y. analyzed the data. M.S., W.Y., S.J., Q.H., Z.Y., S.J. and C.L. wrote the manuscript.

## Conflicts of Interest

The authors declare no conflicts of interest.

## Supporting information




**Supporting File**: advs78562‐sup‐0001‐SuppMat.docx.

## Data Availability

The raw sequence data reported in this paper have been deposited in the Genome Sequence Archive (Genomics, Proteomics & Bioinformatics 2025) in National Genomics Data Center (Nucleic Acids Res 2025), China National Center for Bioinformation / Beijing Institute of Genomics, Chinese Academy of Sciences (GSA: CRA032647, GSA: CRA032498, GSA: CRA032499, GSA: CRA032562) that are publicly available as of the publication date at https://ngdc.cncb.ac.cn/gsa. Further details on data and code availability are provided in the Methods details. All computational codes used in the analysis are available for research purposes and can be provided upon request. Any additional information required to reanalyze the data reported in this paper is available from the lead contact upon request.
