# Supplementary material for: Loss of Lepr in Fibroblasts Drives Tumor Development by Promoting Macrophage Infiltration
Source: Adv Sci (Weinh). 2026 Oct 31:e78562. Online ahead of print. doi: 10.1002/advs.78562 (PMC13723709; doi:10.1002/advs.78562)
Supplement: Supplementary file 1 [file ADVS-9999-e78562-s001.docx]

**Supporting Information**

**Loss of Lepr in fibroblasts drives tumor development by promoting macrophage infiltration**

*Manyu Song*^1,2,^*, *Wuqi Yang*^1,2,^*, *Bangran Qi*^1,2^, *Liyuan Hou*^1,2^, *Xu Yang*^3^, *Jiuzhi Xu*^3^, *Shengyuan Tian*^3^, *Shuiling Jin*^5^, *Lu Yu*^3^, *Zhengquan Yu*^3,4,5^, *Min Deng*^3,#^, *Jiaqiang Huang*^1,2,#^, *Suoqin Jin*^6,#^, *Cong Lv*^1,2,#,§^

**Figure S1** **Lepr-lineage fibroblast -derived Lepr^neg^Mmp^high^ cells are enriched in colon tumors.**

(A) Schematic diagram of the generation of *Lepr-Cre;mTmG* mice.

(B) Schematic diagram of the generation of AOM/DSS-induced tumor mouse model.

(C) Representative Immunofluorescence images of GFP and Col1a1 in AOM/DSS-induced colon tumors from *Lepr-Cre;mTmG* mice. n = 5 mice for each group. Scale bar, 50 μm.

(D) Singlets underwent quality filtering to remove cells with aberrant feature numbers, UMI counts, or excessive mitochondrial gene expression. The remaining high-quality cells were retained for all downstream analyses.

(E) UMAP plot showing the distribution of GFP⁺ fibroblasts from normal colon and tumor tissues of *Lepr-Cre;mTmG* mice. Cells from normal colon and tumor tissues are color-coded for distinction.

(F) Feature plots of expression distribution for GFP.

(G) UMAP plot reveals cellular heterogeneity of GFP⁺ cells from normal colon and tumor tissues of *Lepr-Cre;mTmG* mice. Cells are colored based on the annotated major cell types.

(H) Feature plots of the well-known marker genes of each cell population based on normalized counts. Expression levels are color-coded, with gray indicating low expression and red indicating high expression.

(I) UMAP plot reveals cellular heterogeneity of GFP⁺ fibroblasts from normal colon and tumor tissues of *Lepr-Cre;mTmG* mice. Four distinct clusters are identified and annotated.

(J) In situ hybridization for *Lepr* mRNA and fluorescence for GFP in colon and tumor of *Lepr-Cre;mTmG* mice (n = 3). Scale bar, 50 μm. The proportion of *Lepr*^+^GFP^+^ cells in GFP^+^ cells was quantified.

Data are presented as mean ± SEM (C and J). Unpaired t test (J) was used. **p< 0.01.

Related to Figure 1.

**Figure S2 Abundance and characteristics of an Fb4-like fibroblast population in the broader fibroblast landscape.**

(A) UMAP plot showing cell populations in AOM/DSS-induced mouse colorectal tumors. Cells are colored based on the annotated major cell types.

(B) UMAP plot showing fibroblast heterogeneity after reclustering fibroblasts from the AOM/DSS scRNA-seq dataset.

(C) Dot plot showing the expression of Lepr^neg^Mmp^high^ signature genes across the fibroblast subsets shown in panel B.

(D) Relative abundance of the Fb4-like population among the total fibroblast population shown in panel B.

(E) Dot plot showing the expression of representative apCAF, myCAF, and iCAF marker genes across the fibroblast subsets shown in panel C.

(F) Gene set scoring for inflammatory response pathway in each fibroblast subset in panel B.

Related to Figure 1.

**Figure S3 Identification of a cell population resembling the Lepr^neg^Mmp^high^ fibroblast subset in the human colorectal tumors.**

(A) UMAP showing the distribution of cells from single-cell atlas of human colorectum under both physiological and pathological conditions. Cells from different colorectal datasets are color-coded for distinction. Healthy colorectal tissues (Healthy), uninflamed colorectal tissues (Uninflamed), inflamed colorectal tissues (Inflamed), colorectal polyp tissues (Polyp), adjacent non-tumor colorectal tissues (N), and colorectal cancer tissues (T).

(B) Harmony integration and UMAP visualization analysis of human fibroblasts, eight primary fibroblast clusters (cluster 0, cluster 1, …, cluster 7) were identified. cluster 1 was further subclustered into eight subsets (cluster 1-0, cluster 1-1, …, cluster 1-7) and subsequently reintegrated into the global human fibroblast atlas.

(C) UMAP showing the distribution of fibroblasts from single-cell atlas of human colorectum under both physiological and pathological conditions in panel B.

(D) Gene set scores showing the enrichment of signature genes of Fb1, Fb2 and Fb3 subsets in the human fibroblast single-cell dataset in Figure1H.

(E) Violin plots showing the expression of marker genes for Lepr^neg^Mmp^high^ fibroblasts and hFb4 subset in fibroblasts from Figure1H.

(F) UMAP showing the distribution of fibroblasts from healthy, uninflamed, inflamed, polyp, N, and T tissues in Figure1H.

Related to Figure 1.

**Figure** **S4** **Identification of signaling that promote the transition of Lepr-lineage fibroblasts into Lepr^neg^Mmp^high^ state.**

(A) Feature plots of expression distribution for TNFα receptors (Tnfrsf1a, Tnfrsf1b), TFGβ receptors (Tgfbr1, Tgfbr2, Tgfbr3), VEGF receptors (Flt1, Kdr, Flt4), EGF receptors (Egfr) and Androgen receptors (Ar).

(B) GFP⁺ fibroblasts isolated from *Lepr-Cre;mTmG* mice with or without hypoxia treatment (5% O2 for 48 hours, InvivO2, Baker Ruskinn). qRT-PCR analysis of signature genes of Lepr^neg^Mmp^high^ fibroblast subset (n = 3).

(C) GFP⁺ fibroblasts isolated from *Lepr-Cre;mTmG* mice were cultured with or without Hbegf, TGFα, and Areg treatment for 36 hours. qRT-PCR analysis of signature genes of Lepr^neg^Mmp^high^ fibroblast subset (n = 3).

(D) Dot plot showing expression of Tnf and Tgfb family genes across major cell populations in the AOM/DSS mouse CRC scRNA-seq dataset. Dot size represents the percentage of cells expressing each gene, and color indicates average expression.

(E) CellChat analysis of the Tnf signaling network among major cell populations in mouse CRC. Left, global predicted Tnf signaling network; right, predicted Tnf signaling directed toward fibroblasts. Edge thickness represents predicted communication strength.

(F) Relative contribution of individual Tnf ligand-receptor pairs to predicted Tnf signaling toward fibroblasts in mouse CRC.

(G) CellChat analysis of the Tgfb signaling network among major cell populations in mouse CRC. Left, global predicted Tgfb signaling network; right, predicted Tgfb signaling directed toward fibroblasts. Edge thickness represents predicted communication strength.

(H) Relative contribution of individual Tgfb ligand-receptor pairs to predicted Tgfb signaling toward fibroblasts in mouse CRC.

(I) Dot plot showing expression of TNF and TGF family genes across major cell populations in the human CRC scRNA-seq dataset.

(J) CellChat analysis of the TNF signaling network among major cell populations in human CRC. Left, global predicted TNF signaling network; right, predicted TNF signaling directed toward fibroblasts.

(K) Relative contribution of individual TNF ligand-receptor pairs to predicted TNF signaling toward fibroblasts in human CRC.

(L) CellChat analysis of the TGFb signaling network among major cell populations in human CRC. Left, global predicted TGFb signaling network; right, predicted TGFb signaling directed toward fibroblasts.

(M) Relative contribution of individual TGFb ligand-receptor pairs to predicted TGFb signaling toward fibroblasts in human CRC.

Data are presented as mean ± SEM (B and C). Unpaired t test (B and C) was used. *p< 0.05, **p< 0.01, and ***p< 0.001.

Related to Figure 2.

**Figure S5 Lepr^neg^Mmp^high^ fibroblasts promotes tumor development by promoting macrophage infiltration.**

(A) The module scores of CMS1, CMS2, CMS3 marker genes in different groups in Figure 3B. n = 4 mice for each group.

(B) Comparison of the outgoing (x-axis) and incoming (y-axis) interaction strength of each cell population in AOM/DSS mouse CRC scRNA-seq dataset, with circle size representing the number of significant interactions associated with different cell populations.

(C) Circle plots showing the interaction strength and number of significant ligand-receptor pairs between the Fb4-like subset and other cell subpopulations. Edge width is proportional to the interaction number or strength, and the loops indicate autocrine signaling within the Fb4-like subset.

(D) Dot plot showing representative marker genes used for annotation of major cell populations in the human CRC spatial transcriptomic dataset. Dot size represents the percentage of cells expressing each gene, and color indicates average expression.

(E) UMAP visualization of fibroblasts reclustered into six subpopulations.

(F) Dot plot showing expression of representative Lepr^neg^Mmp^high^/hFb4-associated marker genes across the fibroblast subpopulations.

(G) UMAP visualization of myeloid cells reclustered into macrophage (ST-Macro), monocyte (ST-Mono), and conventional dendritic cell (ST-cDC1) populations.

(H) Dot plot showing representative marker genes used for annotation of the myeloid subpopulations.

Data are presented as mean ± SEM (A). Unpaired t test (A) was used. ns, not significant.

Related to Figure 3.

**Figure S6 hFb4 fibroblast abundance is associated with an inflammation-associated macrophage state in human CRC**

(A) UMAP plot showing macrophage heterogeneity after reclustering macrophage from the human colorectal cancer scRNA-seq dataset.

(B) Dot plots showing the expression patterns of signature genes for each macrophage subset in panel A.

(C) Correlation analysis between the relative abundance of hFb-4 fibroblasts and each macrophage subpopulation across individual human CRC samples. hFb-4 abundance was calculated as the percentage of hFb-4 cells among total fibroblasts, and macrophage-subpopulation abundance was calculated as the percentage of each macrophage subset among total macrophages.

(D) Relative abundance of each macrophage subpopulation in hFb4-low and hFb4-high human CRC samples. Macrophage-subpopulation abundance was calculated as the percentage of each subset among total macrophages. Samples were stratified into hFb-4-low and hFb4-high groups according to the median hFb4 abundance.

(E) Dot plot showing the expression of representative inflammatory and myeloid activation-associated genes across the eight macrophage subpopulations.

(F) KEGG pathway enrichment analysis of Macro_C3 marker genes. The top 10 significantly enriched KEGG pathways are shown.

Data are presented as mean ± SD (D). Wilcoxon Rank-Sum test (D) was used. *p< 0.05 and **p< 0.01.

Related to Figure 3.

**Figure S7. Spatial transcriptomic analysis reveals an association between ST-Fib4 fibroblasts and ST-Macro_C3 macrophages in human CRC**

(A) UMAP visualization of macrophages identified in the human CRC spatial transcriptomic dataset, reclustered into nine subpopulations.

(B) Dot plot showing expression of representative Macro_C3 subset-associated marker genes across the macrophage subsets in the human CRC spatial transcriptomic dataset.

(C) Spatial distribution of ST-Fib4 fibroblasts and ST-Macro_C3 macrophages in two representative human CRC tumor sections. Boxed regions are shown at higher magnification below.

(D) Density distributions of the distance from individual cells of each spatial macrophage subpopulation to their nearest ST-Fib4 fibroblast in three human CRC samples.

(E) Permutation-based spatial proximity analysis showing the percentage of ST-Macro_C3 macrophages located within 50 μm of an ST-Fib4 fibroblast in three human CRC samples. Red vertical lines indicate the observed percentages, and blue distributions represent the expected percentages under the permutation-based null model.

(F) Spatial distribution of the identified cellular niches after further subdivision of macrophages into transcriptionally distinct subpopulations in two representative human CRC tumor sections.

(G) Correlation analysis between the observed-to-expected (O/E) enrichment of ST-Fib4 fibroblasts and ST-Macro_C3 macrophages across spatial niches. Shapes indicate individual samples.

(H) Comparison of ST-Macro_C3 O/E enrichment between ST-Fib4^low^ and ST-Fib4^high^ spatial niches, stratified according to the median ST-Fib4 O/E value.

Data are presented as mean ± SD (H). Wilcoxon Rank-Sum test (H) was used. ***p< 0.001.

Related to Figure 3.

 **Figure S8 Blocking TNFα&TGFβ signaling depressed tumor development**

(A) Schematic diagram of AOM/DSS-induced colon tumor mouse model and inhibitor administration.

(B) Gross images of colon tumors from AOM-DSS mice treated with vehicle (Veh) or dual TNFα/TGFβ pathway inhibitors (AB-Inh). n = 4 mice for each group.

(C) Quantification of tumor number of mice in panel B. n = 4 mice for each group.

(D) Quantification of tumor diameter of mice in panel B. Veh: n = 134 tumors from 4 mice; AB-Inh n = 87 tumors from 4 mice.

(E) Representative Immunofluorescence images of GFP, Il1rl1, and Mmp3 in colon tumors from mice in panel B. Scale bar, 100 μm. The number of GFP^+^Il1rl1^+^Mmp3^+^ cells per mm^2^ was quantified.

(F) Immunohistochemical staining for F4/80 in colon tumors from mice in panel B. Scale bar, 100 μm. The number of F4/80^+^ cells per mm^2^ was quantified.

Data are presented as mean ± SEM (C, D, E and F). Unpaired t test (C, E and F) and Mann-Whitney test (D) were used. ns, not significant, *p< 0.05, **p< 0.01, and ***p< 0.001.

Related to Figure 3.

**Figure S9** **Tcf21-Lepr regulatory axis in fibroblasts mediated macrophage infiltration via Csf3**

(A) Schematic diagram of generation of *Pdgfra-Cre^ERT2^;Lepr^fl/f^* mice.

(B) Schematic diagram of AOM/DSS-induced colon tumor mouse model and tamoxifen administration.

(C) qRT-PCR analysis for *Lepr* in colon tumors from *Lepr^con^* and *Lepr^cko^* mice. n = 5 mice for each group.

(D) Representative Immunofluorescence images of F4/80 and CD206 in colon tumors from *Lepr^con^* and *Lepr^cko^* mice. n = 5 mice for each group. Scale bar, 25 μm. The percentage of CD206⁺ cells within the F4/80⁺ cells was quantified.

(E) qRT-PCR analysis for *Lepr* in colon tumor of *Lepr^con^* and *Lepr^cko^* mice treated with TAM and clodronate liposomes. n = 5 mice for each group.

(F) qRT-PCR analysis for *Lepr* in GFP⁺ fibroblasts treated with siNC, siLepr or siLepr+siCsf3 for 48 hours (n = 3).

(G) qRT-PCR analysis for *Tcf21* in GFP⁺ fibroblasts treated with siNC or siTcf21 for 48 hours (n = 3).

(H) TCGA database analysis showed that *TCF21* expression was downregulated in COAD (Colon adenocarcinoma). Box plots represent the 25th and 75th percentiles, black line indicates median, and whiskers represent extremes. num: sample number of patients.

(I) Schematic diagram of generation of *Pdgfra-Cre^ERT2^;Tcf21^fl/fl^* mice.

(J) qRT-PCR analysis for *Tcf21* in colon tumor from *Tcf21^con^* and *Tcf21^cko^* mice. n = 5 mice for each group.

Data are presented as mean ± SEM (C, D, E, F, H and J). Unpaired test (D, F, G and H) and paired t test (C, E and J) were used. ns, not significant, *p< 0.05, **p< 0.01, and ***p< 0.001.

Related to Figure 4, Figure 5 and Figure 6.
